# Advancing Tuberculosis Antigen Detection by Selecting Sandwich-Compatible Aptamers Against the *Mycobacterium tuberculosis* ESAT-6/CFP-10 Heterodimer Using Library-Immobilized Strategies

**DOI:** 10.1021/acsinfecdis.6c00529

**Published:** 2026-08-31

**Authors:** Shaira Jane Acosta, M. Carmen Bermudo Redondo, Lucie Mužíková Čechová, Marek Ondruš, Vasso Skouridou, Ciara K. O’Sullivan

**Affiliations:** † Interfibio Consolidated Research Group, Departament d’Enginyeria Química, 16777 Universitat Rovira i Virgili, 43007 Tarragona, Spain; ‡ Institute of Organic Chemistry and Biochemistry, Czech Academy of Sciences, Flemingovo nam. 542/2, 160 00 Prague 6, Czech Republic; § Institució Catalana de Recerca i Estudis Avancats (ICREA), 08010 Barcelona, Spain

**Keywords:** tuberculosis, Mycobacterium tuberculosis, aptamers, bait-SELEX, capture-SELEX, ESAT-6/CFP-10 heterodimer

## Abstract

Tuberculosis (TB) represents the world’s leading infectious disease killer, surpassing COVID-19. Despite being preventable and curable, a substantial proportion of cases remain undiagnosed or unreported, sustaining transmission. The detection of antigens such as the *Mycobacterium tuberculosis* ESAT-6/CFP-10 heterodimer, which are secreted at different stages of infection, is a promising screening strategy. Traditional antigen tests relying on antibodies suffer from significant problems of specificity and sensitivity. Aptamers, selected in an *in vitro* technique termed SELEX (**S**ystematic **E**volution of **L**igands by **EX**ponential enrichment), are short, single-stranded oligonucleotides that fold into unique 3D structures, allowing them to bind to specific targets, including proteins, cells, or small molecules, with high affinity and specificity. However, conventional SELEX methods often yield suboptimal aptamers partly due to target immobilization that can promote nonspecific interactions and distort protein conformation. Here, we employed two library-immobilized strategies to generate aptamers against the *M. tuberculosis* ESAT-6/CFP-10 heterodimer. We first performed capture-SELEX to select aptamers against the unconstrained, conformationally native heterodimer target in solution. These selected aptamers were then exploited in a second SELEX, where aptamer-target complexes were “baited” by a target-specific antibody to isolate sandwich-compatible aptamers for downstream assay development. The selected aptamers exhibited high nanomolar affinity regardless of library and selection strategy employed, with bait-SELEX-derived candidates emerging after fewer selection rounds. These aptamers were successfully applied in antibody/aptamer hybrid sandwich assays, supporting their potential for TB antigen detection in nonsputum samples.

## Graphical abstract

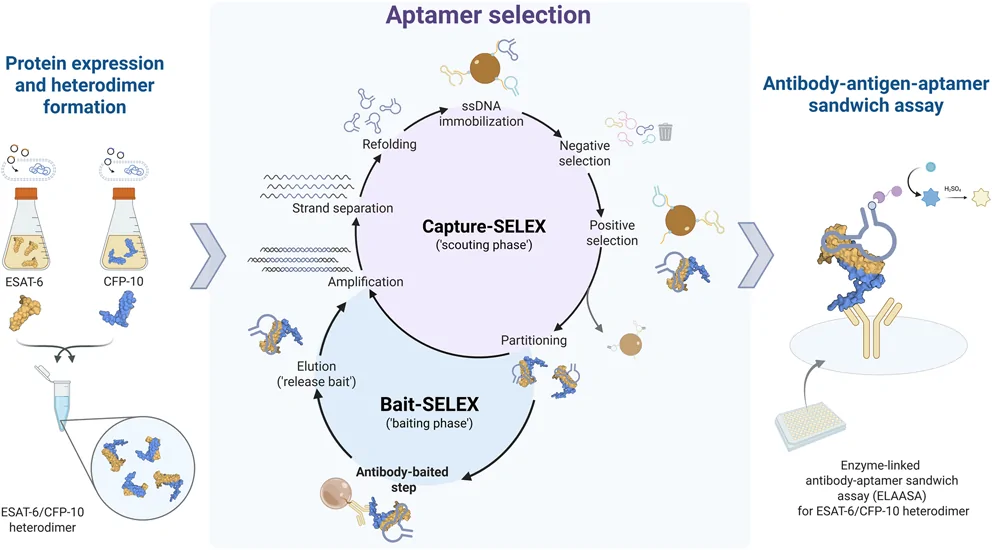

## Introduction

Nearly one in four of the world’s population is infected with tuberculosis (TB).^1^ Transmission of TB occurs via droplet aerosols containing *Mycobacterium tuberculosis*, a bacterium responsible for the infection.^2^ It is estimated that about 5–10% of those infected with TB will manifest clinical symptoms and develop active disease,^1^ with the risk of progression increasing in the presence of comorbidities such as human immunodeficiency (HIV) infection, malnutrition, diabetes, smoking, and poverty.^3–5^ Despite being both preventable and treatable, TB remains the leading cause of death by a single infectious agent, with about 10.7 million people infected with TB in 2024, and resulting in 1.23 million deaths from the disease.^1^ Significant gaps persist between estimated TB incidence and reported cases driven partly by healthcare access limited to fewer than 50% of the population,^1^ contributing substantially to the overall disease burden.

TB exists in a spectrum of continuum asymptomatic infection states that can potentially reactivate and progress to symptomatic active disease.^6^ While *M. tuberculosis* primarily affects the lungs, it is multisystemic in nature.^7^ Pulmonary TB (PTB), a disease that can develop at various stages, often manifests gradually, but a more acute onset of symptoms may be observed in children and immunocompromised individuals.^8^ Typically, PTB presents with fever, night sweats, weight loss, and persistent cough, which can progress to lasting sequelae.^9,10^ It represents a predominant clinical manifestation that accounts for about 80% of the cases, although extrapulmonary TB (EPTB) is also commonly encountered.^1,11^ The heterogeneity of TB often impedes detection, especially during the early stages of infection when clinical manifestations are minimal.^12^

TB diagnostic methods have advanced substantially, yet conventional approaches still predominate in high-burden areas. TB infection is typically identified by the host’s immune response to *M. tuberculosis*-specific antigens using tuberculin skin tests (TST)^13^ or interferon-γ release assays (IGRAs),^14^ whereas active disease is diagnosed through microbiological culture, the gold standard to confirm viable *M. tuberculosis* cells.^15^ Sputum smear microscopy for acid-fast bacilli is usually the primary method used in low-resource settings as it is rapid and low cost.^16^ However, these methods have drawbacks that can impede case identification as they may either have long turnaround times or exhibit poor sensitivity and specificity.^17,18^ While the advent of molecular-based methods, such as Xpert MTB/RIF (Cepheid), has improved TB detection, their high cost and infrastructure requirements limit their use in resource-poor settings, therefore posing considerable challenges in high TB-burden countries.^19^

To address these gaps, rapid point-of-care (PoC) tests have emerged as attractive alternatives to laboratory-based methods. Specifically, immunochromatographic (IC) strip tests have been developed to detect TB biomarkers including lipoarabinomannan (LAM)^20^ and antigen 85B (Ag85B)^21^ in urine. Sandwich-based assays for the detection of antigens are robust and have been widely used in many platforms, including lateral flow assays and electrochemical biosensors. While antibodies can provide high sensitivity, they have inherent limitations such as high cost, time-consuming animal-based production, and challenges associated with labeling and modification.^22,23^ These limitations have spurred the development of alternative biorecognition molecules for integration in PoC platforms, particularly nucleic acid–based biorecognition molecules. For instance, nucleic acid aptamers have been valued for their programmability, rapid synthesis, and adaptability to bind diverse targets^24^ and their small size and flexibility to integrate in different assay formats for rapid diagnostics,^25^ including lateral flow assays, displacement assays,^26,27^ and electrochemical biosensors and fluorescent and molecular aptamer beacons.^28^

Aptamers are nucleic acid–based biorecognition molecules capable of folding into specific three-dimensional (3D) structures to bind specifically to their cognate target.^29^ They offer advantages including cost-effective and scalable synthesis, facile chemical modification, and adaptability to different biosensing and diagnostics platforms.^30,31^ Aptamers are isolated using a library selection method called Systematic Evolution of Ligands by EXponential enrichment (SELEX).^32^ SELEX is performed in iterative rounds of binding, partitioning, and amplification.^31,33^ Many variants of SELEX have been developed since its inception.^34–37^ Conventionally, SELEX involves the immobilization of targets on a solid support while the aptamer library remains in solution. However, immobilization can significantly alter the target’s structural conformation, potentially leading to the selection of aptamers that recognize non-native conformations.^38^ Library-immobilized SELEX variants have also been reported.^39–42^ In principle, the aptamer library is immobilized on a solid support via hybridization with a partially complementary probe. When exposed to the free native target, sequences undergo significant conformational changes to enable target–aptamer binding in solution, while the nonbinding sequences remain tethered. This strategy is especially advantageous for selecting aptamers against targets challenging to immobilize.^43^

To date, the incorporation of aptamers in TB antigen tests remains limited, largely attributable to challenges in selection and the lack of standardized aptamer-based diagnostic platforms.^44^ The *M. tuberculosis* early secretory antigenic target 6 kDa (ESAT-6) and the culture filtrate protein 10 kDa (CFP-10) antigens have significant diagnostic values in nonsputum samples.^45–48^ ESAT-6 and CFP-10 are secreted proteins essential for *M. tuberculosis* pathogenesis.^47,49^ CFP-10 forms a stable 1:1 heterodimer complex with ESAT-6, the major virulence factor,^50^ after secretion,^51^ and later dissociates from the complex under acidic conditions during phagosomal escape.^52,53^ Encoded within the region of difference 1 (RD1) gene, which is deleted in the Bacillus Calmette-Guérin (BCG) vaccine,^54^ these proteins regulate macrophage cell death at various stages of TB infection.^47,55,56^ To date, two selections have targeted the ESAT-6/CFP-10 heterodimer. The first employed filter membrane SELEX and a heterodimeric complex formed *in vitro* by mixing the two monomeric ESAT-6 and CFP-10 proteins. Aptamers with low-nanomolar affinity were obtained, but binding was restricted to the CFP-10 subunit.^57^ The second one used microplate-based SELEX against a single-chain recombinant ESAT-6.CFP-10 fusion, reporting aptamers with high nanomolar affinity.^58^ Multimeric targets pose unique challenges for aptamer selection, as conventional methods rely on immobilization on solid supports, which can be difficult for conformationally dynamic targets such as the ESAT-6/CFP-10 heterodimer.^59–61^

In this work, we introduce a library-immobilized strategy pipeline for the selection of aptamers against the *M. tuberculosis* ESAT-6/CFP-10 heterodimer to develop biorecognition molecules for antigen-based biosensing tools in a sandwich format. We employed capture-SELEX^42^ as the first step for pre-enrichment of aptamers against the unconstrained state of the heterodimeric target. Building upon this, in a process, we term “bait-SELEX,” a strategy was designed to pull down aptamer/target complexes, capable of forming antibody/target/aptamer sandwich complexes using a target-specific antibody immobilized on magnetic beads. Two ssDNA libraries with distinct architectures were used in parallel in the two SELEX modalities, and enrichment and evolution were monitored throughout the selection processes. Candidate aptamers identified by next-generation sequencing (NGS) were screened and evaluated using biochemical and biophysical assays. Finally, a proof-of-concept enzyme-linked antibody/aptamer hybrid sandwich assay (ELAASA) was demonstrated in which the selected aptamers served as reporter probes.

## Results and Discussion

### Preparation of Recombinant ESAT-6 and CFP-10 Proteins and of Their Heterodimer

In working toward aptamer selection against the *M. tuberculosis* ESAT-6/CFP-10 heterodimer, recombinant ESAT-6 and CFP-10 proteins were individually expressed from their respective plasmids in *E. coli* and purified with Immobilized Metal Affinity Chromatography (IMAC). High-purity proteins (>90%) with the expected size were obtained (Figure 1a and Figure S1a) and recognized by their respective polyclonal antibodies (Figure S1b). The heterodimer was prepared by incubating an equimolar mixture of the two proteins in neutral pH solution at ambient temperature. As can be seen in Figure 1b, native PAGE analysis revealed a distinct band of intermediate mobility absent in the individual proteins, while no band corresponding to the individual proteins could be observed. While the two proteins are almost identical in size, the calculated isoelectric point of ESAT-6 based on its expected amino acid sequence is slightly lower compared to CFP-10 (5.18 vs 5.72), resulting in higher mobility under native electrophoretic conditions. On the other hand, the ESAT-6/CFP-10 complex has an intermediate charge, consistent with its intermediate mobility compared to the individual proteins. These results confirm the formation of the heterodimer, in agreement with a previous report.^59^ Intrinsic tryptophan fluorescence spectroscopy further validated the complex assembly, as the blue-shifted emission peaks from 347–351 nm for the individual proteins to 341 nm for the heterodimer indicated reduced solvent accessibility of tryptophan residues upon dimerization (Figure 1c). When the mixture of the two proteins was analyzed without prior incubation, an emission maximum at 346 nm was observed, which may suggest incomplete heterodimer formation. Overall, these results demonstrate the successful *in vitro* formation of the ESAT-6/CFP-10 heterodimer and are consistent with previous reports detailing the preparation and successful use of the formed complex in functional studies.^59,60,62^

**Figure 1:**
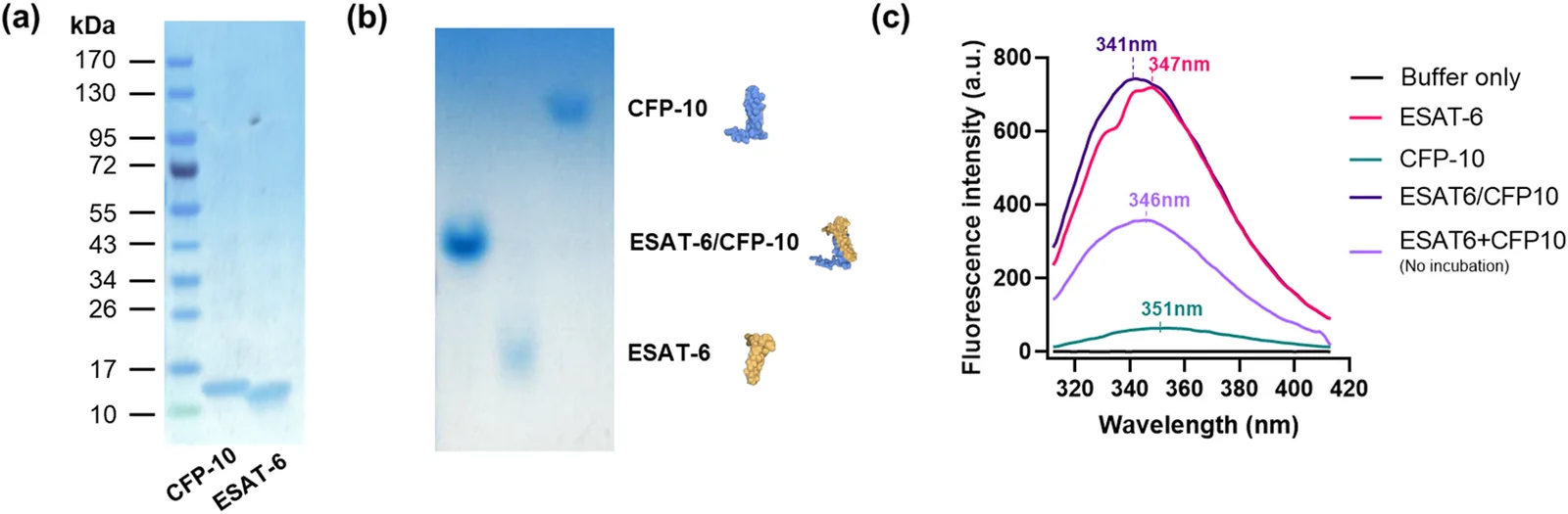
*M. tuberculosis* ESAT-6/CFP-10 heterodimer complex formation. (a) SDS-PAGE of purified recombinant ESAT-6 and CFP-10. (b) Native PAGE analysis of the individual proteins before and after complex formation using an equimolar mixture of the two proteins. (c) Intrinsic tryptophan fluorescence emission spectra of the two proteins individually and of their mixture with no incubation and after 2 h incubation at ambient temperature.

### Development of Pipeline for Library-Immobilized Aptamer Selections for ESAT-6/CFP-10 Heterodimer Protein

The library-immobilized SELEX approaches employed for the discovery of aptamers against the *M. tuberculosis* ESAT-6/CFP-10 heterodimer protein are summarized in Figure 2. These were capture-SELEX and a novel strategy termed bait-SELEX. In capture-SELEX, the ssDNA library was immobilized on magnetic beads via hybridization through a short, partially complementary probe, while the target protein remained in solution. This selection format prevents the structural distortion of the target protein and aims to preserve the heterodimer conformation without orientation bias. Moreover, it can potentially minimize nonspecific binding to solid supports that often occurs with target immobilization.^37,62,63^ To facilitate the selection of aptamers that can be used in sandwich assays, bait-SELEX, an antibody-assisted selection step, was introduced. Specifically, the aptamer-target complexes formed in solution during capture-SELEX (scouting phase) were captured by an anti-CFP-10 polyclonal IgG antibody immobilized on magnetic beads (baiting phase). The goal of bait-SELEX was to isolate aptamers binding to the heterodimeric target for their downstream combination with the anti-CFP-10 antibody in sandwich assay formats for biosensing applications. Aptamer selection for the heterodimer was completed in 11 cycles of capture-SELEX and 6 cycles of bait-SELEX, with each round requiring roughly a typical laboratory day to complete binding, partitioning, and PCR and ssDNA preparation.

**Figure 2:**
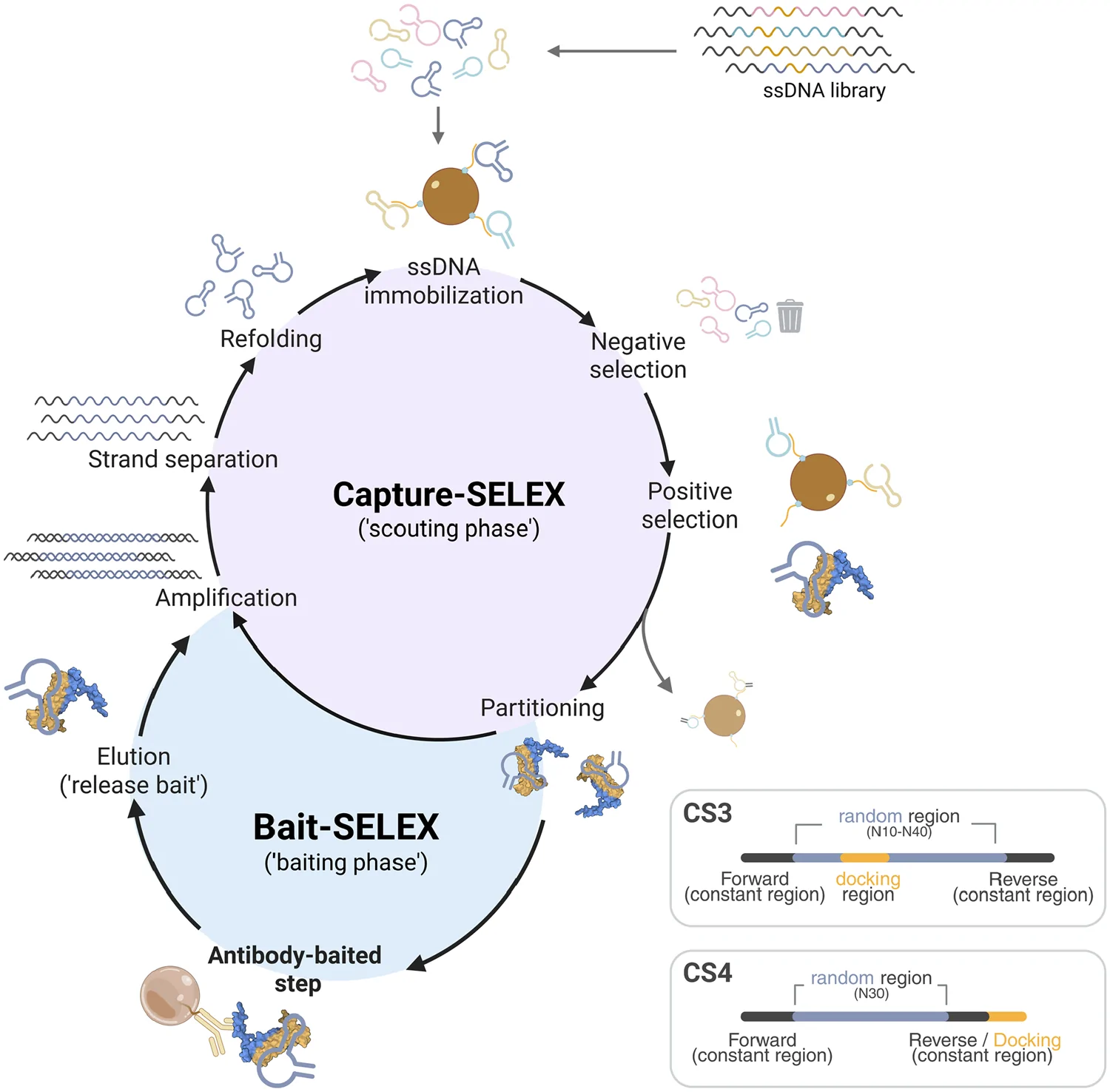
Overall pipeline for library-immobilized SELEX approaches against *M. tuberculosis* ESAT-6/CFP-10 heterodimer. In capture-SELEX, the immobilized ssDNA sequences displace to the solution to bind the target, forming aptamer/target complexes. Bait-SELEX consists of two phases: scouting of aptamers by capture-SELEX and baiting of aptamer-target complexes with an anti-CFP-10 antibody immobilized on magnetic beads. This allows the isolation of aptamers capable of forming sandwich complexes with the target and the antibody.

Two structurally distinct aptamer libraries were employed in parallel selections, each flanked with different constant primer-binding regions. CS3 library (98 nt) contained two random regions of 10 nt and 40 nt separated by a constant 12-nt docking site, complementary to a capture probe immobilized on magnetic beads.^42^ The CS4 library was shorter (66 nt) and contained a single 30-nt random region (N30), while a 12-nt contiguous segment of the reverse primer-binding region served as the docking site. Capture-SELEX was carried out by immobilizing the libraries on streptavidin-magnetic beads via hybridization through their respective biotinylated capture probes, which were different to each other to avoid contamination. Once immobilized, weakly bound ssDNA sequences were removed during a short pre-elution step in binding buffer at a slightly elevated temperature (28 °C). A negative selection step was then carried out in binding buffer (background elution), followed by positive selection during which a high concentration of the target in binding buffer was added to the immobilized libraries. If the sequences had higher binding affinity for the target than their respective complementary docking sequences, they were released from the magnetic beads^63,64^ and magnetically separated. This fraction, containing the target/aptamer complexes, was used for amplification and ssDNA generation for succeeding rounds of selection. To enhance the binding properties of the selected aptamers, stringency was increased during the SELEX process. This included decreasing the amount of the ssDNA pools used to immobilize on the magnetic beads, the duration of the incubation of the pools with the target as well as the concentration of the target (Table S2). A total of 11 rounds of capture-SELEX were carried out. The first four rounds of capture-SELEX also served as the scouting phase for bait-SELEX, a strategy designed to enrich aptamers capable of binding the ESAT-6/CFP-10 heterodimer target simultaneously with an anti-CFP-10 IgG antibody, suitable for a sandwich assay format. To achieve this, the target-elution fraction from capture-SELEX was incubated with an anti-CFP-10 IgG antibody immobilized on magnetic beads (detailed in the Supporting Information and Figure S2). Target/aptamer complexes that could be simultaneously bound by the antibody as a bait were pulled down by the beads, separated magnetically from the unbound complexes, and eluted with glycine. These eluted sequences were then amplified and used for another two-step bait-SELEX round—scouting of target-binding sequences with capture-SELEX and antibody-baiting steps for selecting sandwich-forming ones. Bait-SELEX was carried out for a total of 6 rounds, with the last two rounds only involving the antibody–bait step.

### Monitoring of the Selection Progress and Pool Diversity

The progress of the selections was evaluated by pilot PCR, qPCR, and melt curve analysis (Figure 3). Pilot PCR was carried out at the end of each round by amplifying recovered ssDNA from elution fractions. Analysis of the PCR reactions by agarose gel electrophoresis showed that increasing selection stringency resulted in reduced nonspecific background elution, which are sequences displacing in the absence of any target, thus enriching target-binding sequences in the target-elution fractions. Enrichment of target-bound sequences was evident as early as round three, regardless of the starting library. For the bait-SELEX scouting phase (rounds 1–4), most preformed aptamer/target complexes were not captured by the anti-CFP-10 antibody on magnetic beads, as indicated by the faint amplicon bands in the antibody-elution fractions (“AE” wells) compared to unbound complexes in solution (“S” wells). This finding was not surprising, since the initial libraries were unlikely to contain an abundance of aptamer sequences binding nonoverlapping epitopes with the antibody. Nevertheless, after two rounds of bait-SELEX, faint bands appeared in the antibody-elution fractions, particularly in the selection with the CS3 library, suggesting emergence of sandwich-compatible aptamers.

**Figure 3:**
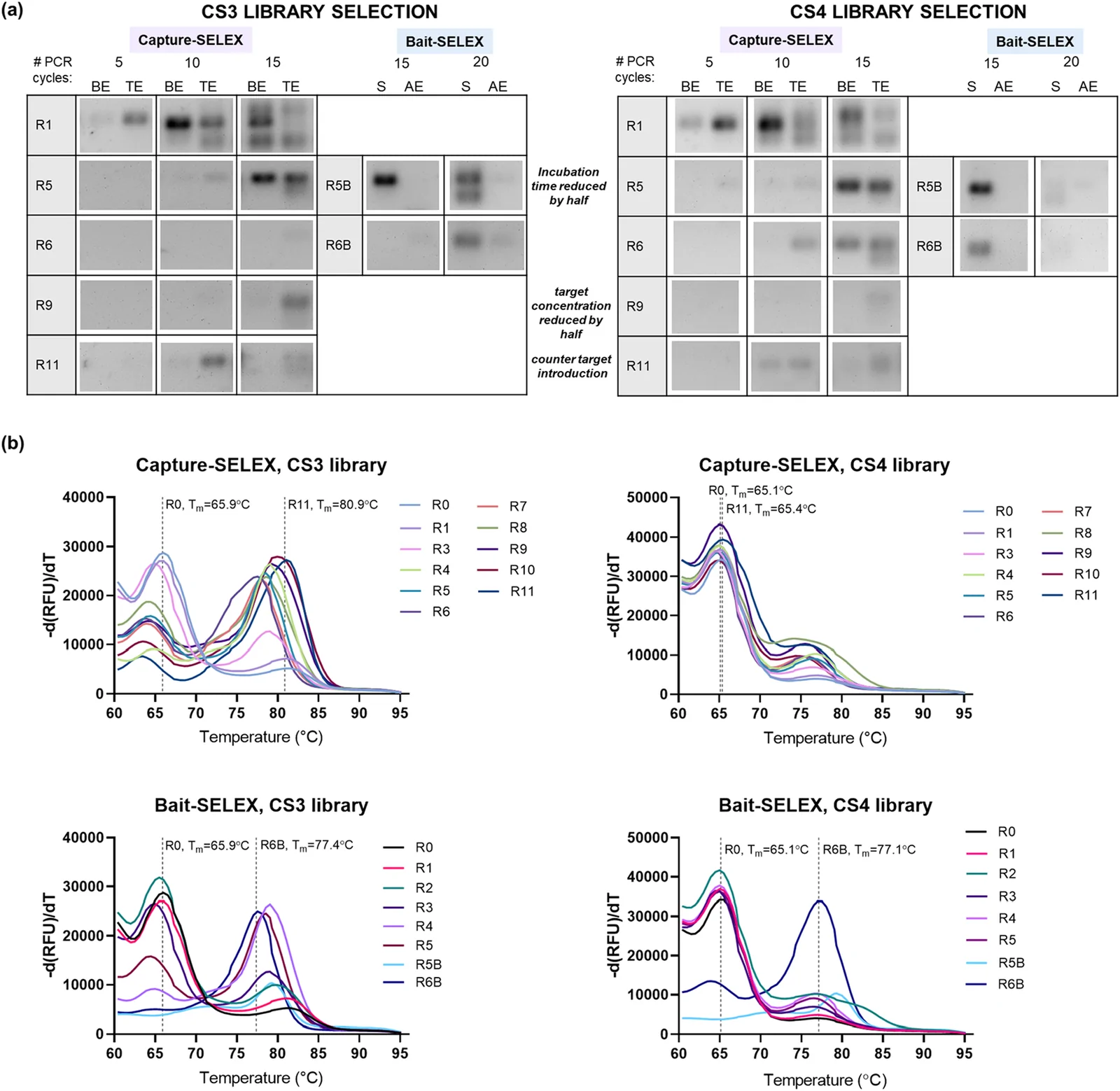
Progress of the capture-SELEX and bait-SELEX strategies. (a) Pilot PCR with target-bound recovered ssDNA from each selection round to assess amplification efficiency. The products were analyzed by agarose gel electrophoresis. BE: background elution (buffer only); TE: target elution; S: supernatant after antibody–bait step in bait-SELEX; AE: elution of antibody-bound aptamer-target complexes in bait-SELEX. (b) Melting profiles of aptamer pools to assess sequence diversity. The eluted ssDNA from each round was amplified by qPCR and the melting patterns of the corresponding dsDNA pools were analyzed. -d­(RFU)/dT: negative first derivative of the fluorescence signal against the temperature.

The recovery of ssDNA at the end of each round was evaluated by qPCR. Specifically, the amount of ssDNA in the capture-SELEX target-elution fractions, as well as in the bait-SELEX antibody-elution fractions, was quantified by qPCR and normalized to the input ssDNA amount for each round to calculate the recovery rates. High recovery rates were observed in the first three rounds, which were common to both capture-SELEX and bait-SELEX in the selection with the CS3 library, whereas lower recovery was observed when the CS4 library was used (Figures S3 and S4). The two libraries differ primarily in the mode of immobilization on the magnetic beads and the lengths of their random regions. The CS3 library was immobilized through partial hybridization to an internal docking site located between the two random regions. On the other hand, the CS4 library was tethered via a region of the reverse primer-binding site, slightly distanced from the sole random region. As a result, the two immobilization strategies create different spatial constraints after hybridization, which may affect the sequence displacement and target binding. Furthermore, increasing stringency by halving the concentration of the target in round four led to a marked decrease in recovery. Nevertheless, substantial recovery was observed in the final selection round 11 with capture-SELEX (Figure S3). With regard to bait-SELEX, introduction of the antibody–bait step in the fifth round also eliminated a substantial fraction of the pool, as expected, since only those target-bound aptamers capable of forming a sandwich with the antibody could be retained from the target-elution pools (Figure S4). However, after the completion of a second bait-SELEX round, increased ssDNA recovery was observed, indicating enrichment of antibody sandwich-forming aptamers, especially in the selection carried out with the CS3 library. In contrast, ssDNA recovery during the baiting phase was substantially lower in the CS4 pools, suggesting reduced enrichment efficiency or weaker retention of target-binding sequences, particularly those capable of forming a sandwich with the antibody.

The diversity of the aptamer pools was assessed by analyzing their melt curve profiles (Figure 3b). A shift in the melt temperature (*T*_m_) was observed for the CS3-derived pools, from approximately 66 °C during the initial three rounds of capture-SELEX to progressively higher temperatures (77–81 °C) in later rounds, consistent with a change in pool composition and enrichment in more stable sequences with higher melting temperatures (higher %GC). The increasing sharpness of the melting peaks as selection progressed further indicated higher homogeneity and enrichment of the aptamer pools. In contrast, when the CS4 library was used, no significant differences were observed in the melting temperatures of the pools from the different stages of the selection, indicative of significantly lower enrichment and pool diversity. This is consistent with the markedly reduced ssDNA recovery observed in capture-SELEX performed with the CS4 library as compared to CS3 (Figure S3). Moreover, in the selection with CS4, no clear melting temperature transition was observed as the SELEX progressed, but the final round showed an apparent high-temperature melt peak accompanied by a broad low-temperature shoulder. The melting profiles of the CS3 aptamer pools in both capture-SELEX and bait-SELEX are in agreement with previous reports,^65^ where highly diverse mixtures from each round across a temperature range of 60–95 °C showed a progressive shift in the average melting temperature as SELEX progressed. In contrast, for the CS4 in bait-SELEX, a shift in the melting peak only emerged at the final stage of the selection suggesting that sequence convergence may have been incomplete.

The superior performance observed with the CS3 library may be attributed to its longer, segmented random region (10 nt and 40 nt, separated by the docking site), which likely provided greater conformational flexibility for enriching target-binding aptamers. Conversely, the CS4 library’s continuous 30-nt random region offered limited sequence space for complex structural evolution. Although 30-nt nonsegmented libraries have been successfully reported previously in capture-SELEX for small molecules,^66,67^ the CS4 was less efficient in this work for the *M. tuberculosis* ESAT-6/CFP-10 heterodimeric complex. This reduced enrichment aligns with reports emphasizing the importance of the length of the random region. For example, Nutiu and Li^39^ showed that a 30-nt random region library failed to yield aptamers against CTP/UTP due to insufficient diversity and structural flexibility for stable binding. This may explain the comparatively poor performance of the CS4 library in enriching the aptamer pools and that potentially high-binding affinity aptamers only appear in the latter rounds of the selection. The CS3 library’s design, resembling the successful BANK-S4 library used by Stoltenburg et al.^42^ in capture-SELEX, likely enhanced structural diversity and interactions with the complex target, yielding stronger binding. Nonetheless, limitations in the immobilized library’s quantity and diversity may also have influenced the overall selection efficiency.

### NGS Data Analysis and Identification of Aptamer Candidates

High-throughput Ion Torrent Next-Generation Sequencing (NGS) was carried out to identify enriched aptamers in selected pools. The resulting data sets were processed to determine the number of reads (sequences) with library length, the unique fraction and the enrichment achieved following previous workflow.^68^ As can be seen in Figure 4a, increasing enrichment was observed when capture-SELEX was carried out using the CS3 library, with enriched sequences rising from approximately 4% in round four to almost 60% by the end of the selection (round 11). In contrast, enrichment with the CS4 library was only observed at the end of the selection, reaching about 20% in the last round. Melt curve analysis (Figure 3b) supported this enrichment patterns between CS3 and CS4 selections, despite the comparable GC content (47–52%) across the selection pools. These results indicate that the increased sequence diversity resulting from the more complex CS3 library design was more suitable for the selection of aptamers against the *M. tuberculosis* ESAT-6/CFP-10 heterodimer. The simpler CS4 library design based on a single shorter random region flanked by primer annealing sites, was considerably less efficient. In bait-SELEX, we also assessed the overall enrichment by monitoring the reduction in unique sequence diversity and the corresponding increase in enriched sequence species across selection rounds. Notably, while pools from the scouting phase (R3 and R4) in both the CS3 and CS4 selections showed minimal enrichment (Figure 4b), a marked enrichment was observed during the baiting phase, which can be attributed to the increased stringency imposed by the antibody-assisted isolation of sequences capable of forming sandwich complexes. A progressive decrease in the average GC content of the aptamer pools from 52% and 50% in the initial CS3 and CS4 libraries, respectively, to 46% in the final enriched pools was observed. This trend of decreasing GC content indicates a selection for AT-rich sequences, which are expected to have lower thermal stability.^65^

**Figure 4:**
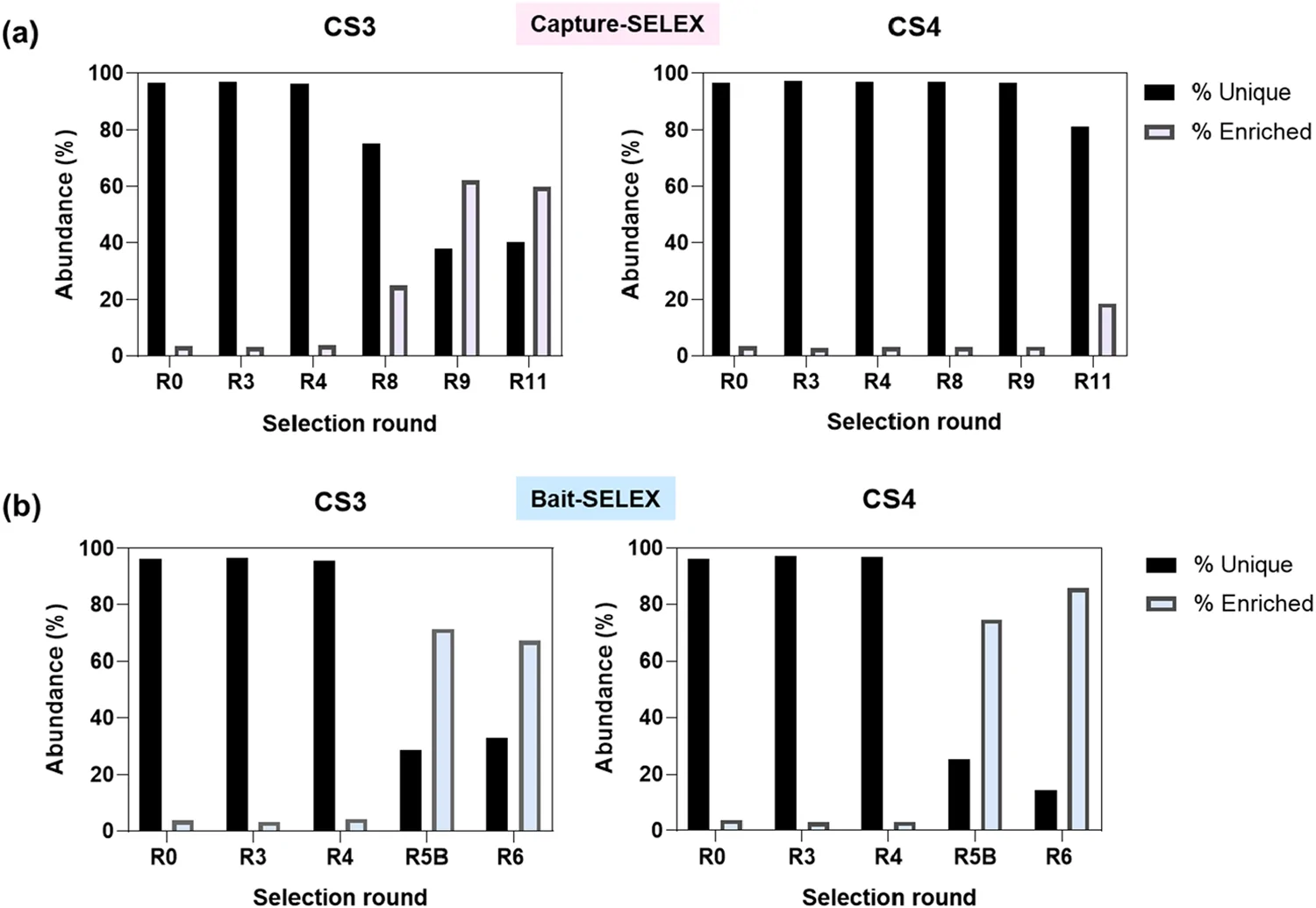
Enrichment of the selections evaluated by Next-Generation Sequencing (NGS) and bioinformatic analysis. The unique and enriched sequences in the selected pools were calculated for each selection carried out with CS3 and CS4 libraries employing (a) capture-SELEX and (b) bait-SELEX.

A total of 17 representative aptamer candidates were finally selected from both capture-SELEX and bait-SELEX for further analysis (Table S3). Candidates were first chosen based on their preferential high abundance in the target-elution pools relative to the respective negative selection pools in the final round of each selection. Subsequently, representative sequences were selected from distinct sequence families identified with the Clustal Omega web tool, prioritizing those with higher predicted structural stability (Δ*G* values calculated with the Mfold web tool). These included ten sequences from capture-SELEX (three from the CS3 and seven from the CS4 selections) and seven sequences from bait-SELEX (three from the CS3 and four from the CS4 selections). No sequence motifs were identified after analysis with the MEME suite. The secondary structures of the selected candidate aptamers were predicted using Mfold. As can be seen in Figures S5 and S6, CS3-derived aptamers from capture-SELEX and bait-SELEX maintained a randomized core region that was either structurally independent or minimally involved in hairpin loop structures with the 18-nt flanking primer-binding sites. Conversely, CS4-derived aptamers from both selection strategies consistently integrated a partial involvement of the primer regions in overall folding. This suggests that shorter and less complex sequences may promote more compact secondary structures in which both primer-binding sites participate in aptamer structures, potentially influencing amplification efficiency and progress of selection.^38^

### Screening of Aptamer Candidates

An enzyme-linked aptamer assay (ELAA) was employed to screen the aptamer candidates and evaluate their binding to the ESAT-6/CFP-10 heterodimer immobilized in the wells of a microtiter plate. Among the capture-SELEX candidates, CS3-derived HC309p and CS4-derived HC4133p aptamers exhibited the highest binding signals (Figure 5a). Bait-SELEX aptamers generally produced higher signals than those obtained from capture-SELEX (Figure 5b), with the highest sensitivity observed when aptamers HP301p (CS3 library), and HP414p, HP419p, and HP429p (CS4 library) were employed. Bait-SELEX candidates were further evaluated in a sandwich assay format designed to mimic SELEX conditions and confirm their capacity to form sandwiches with the anti-CFP-10 antibody used during selection. In this assay, the same antibody was immobilized on the microtiter plate wells, and the ESAT-6/CFP-10 heterodimer captured by the antibody was detected using individual aptamer candidates. As shown in Figure 5c, while all candidates could form a sandwich complex with the antibody-captured target, aptamers HP301p and HP412p generated the highest signals. Based on their sensitivity in detecting the ESAT-6/CFP-10 heterodimer, HC309p and HC4133p from capture-SELEX, as well as HP301p and HP412p from bait-SELEX, were finally selected for further characterization. For bait-SELEX candidates, the selection also considered their ability to form an effective sandwich with the anti-CFP-10 antibody and enable sensitive detection of the heterodimer.

**Figure 5:**
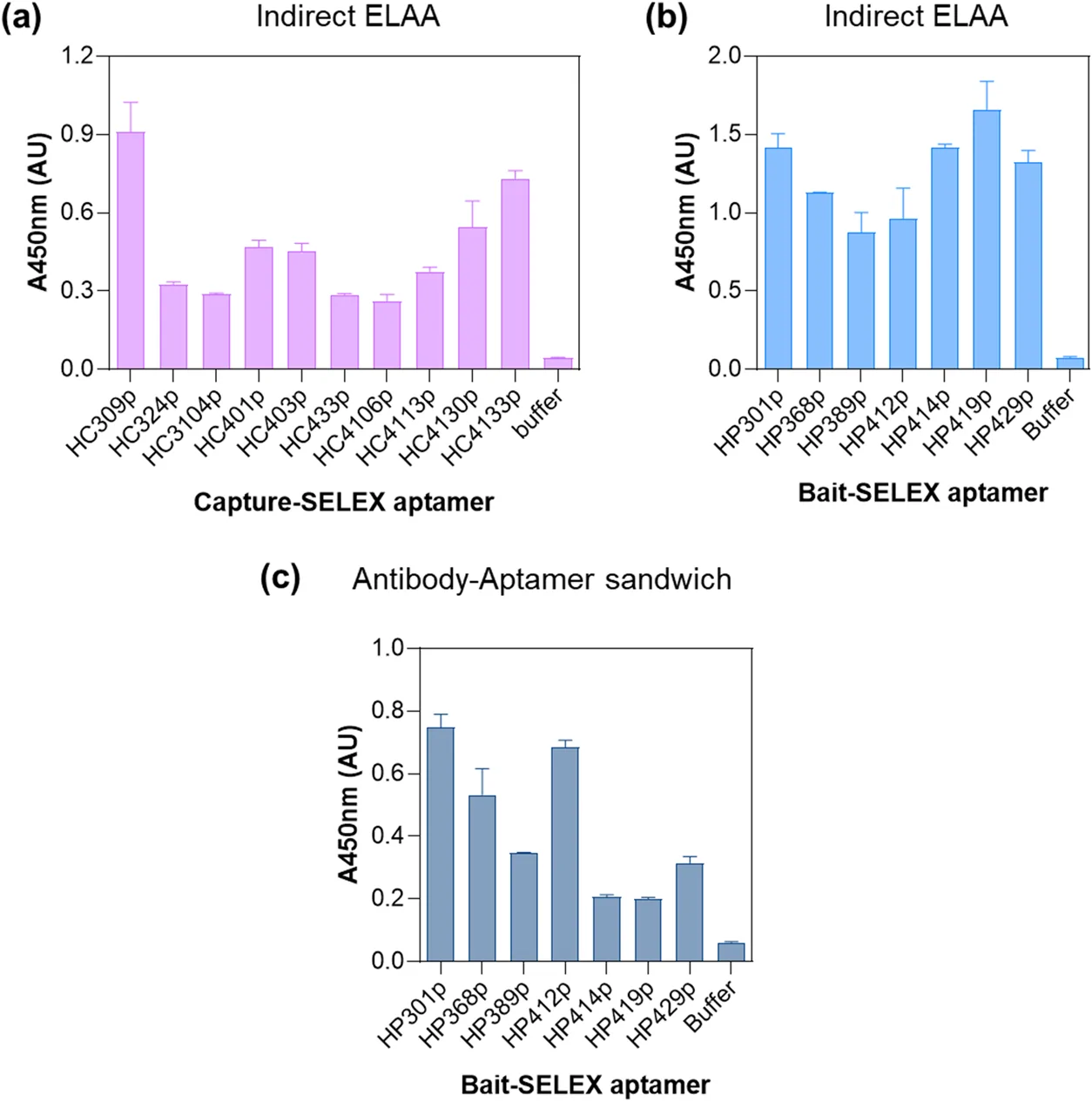
Evaluation of the binding of the aptamer candidates to *M. tuberculosis* ESAT-6/CFP-10 heterodimer. Indirect enzyme-linked aptamer assay (ELAA) for screening the candidates from (a) capture-SELEX and (b) bait-SELEX. (c) Antibody–aptamer sandwich assay to screen the bait-SELEX candidates. The anti-CFP-10 antibody employed during the selection was used to capture the heterodimer and each aptamer candidate to detect the complex. All aptamers were biotinylated at their 5′-end and used in combination with a streptavidin-polyHRP (SA-pHRP) enzyme conjugate.

### Determination of the Binding Affinities of Aptamers to ESAT-6/CFP-10 Heterodimer

Indirect ELAA and biolayer interferometry (BLI) were used to monitor aptamer-target protein interactions and determine the binding affinity of the selected aptamers. In ELAA, the target protein was immobilized on the surface of a microplate well, and different concentrations of the biotinylated aptamers were used in solution. BLI measurements were carried out using immobilized biotinylated aptamers on streptavidin-coated biosensors, while different concentrations of the target protein were in solution. Employing these two complementary assay formats reinforces the reliability of the results and minimizes discrepancies arising from artifacts such as heterogeneous surface density or reduced binding site accessibility.^69^ As shown in Figure 6, the *K*_D_ values calculated by indirect ELAA were in the nanomolar range. Specifically, aptamers HC309p and HC4133p from capture-SELEX exhibited *K*_D_ values of 100.2 and 273.4 nM, respectively, while for bait-SELEX aptamers HP301p and HP412p, the calculated *K*_D_s were 232.3 and 91.8 nM, respectively.

**Figure 6:**
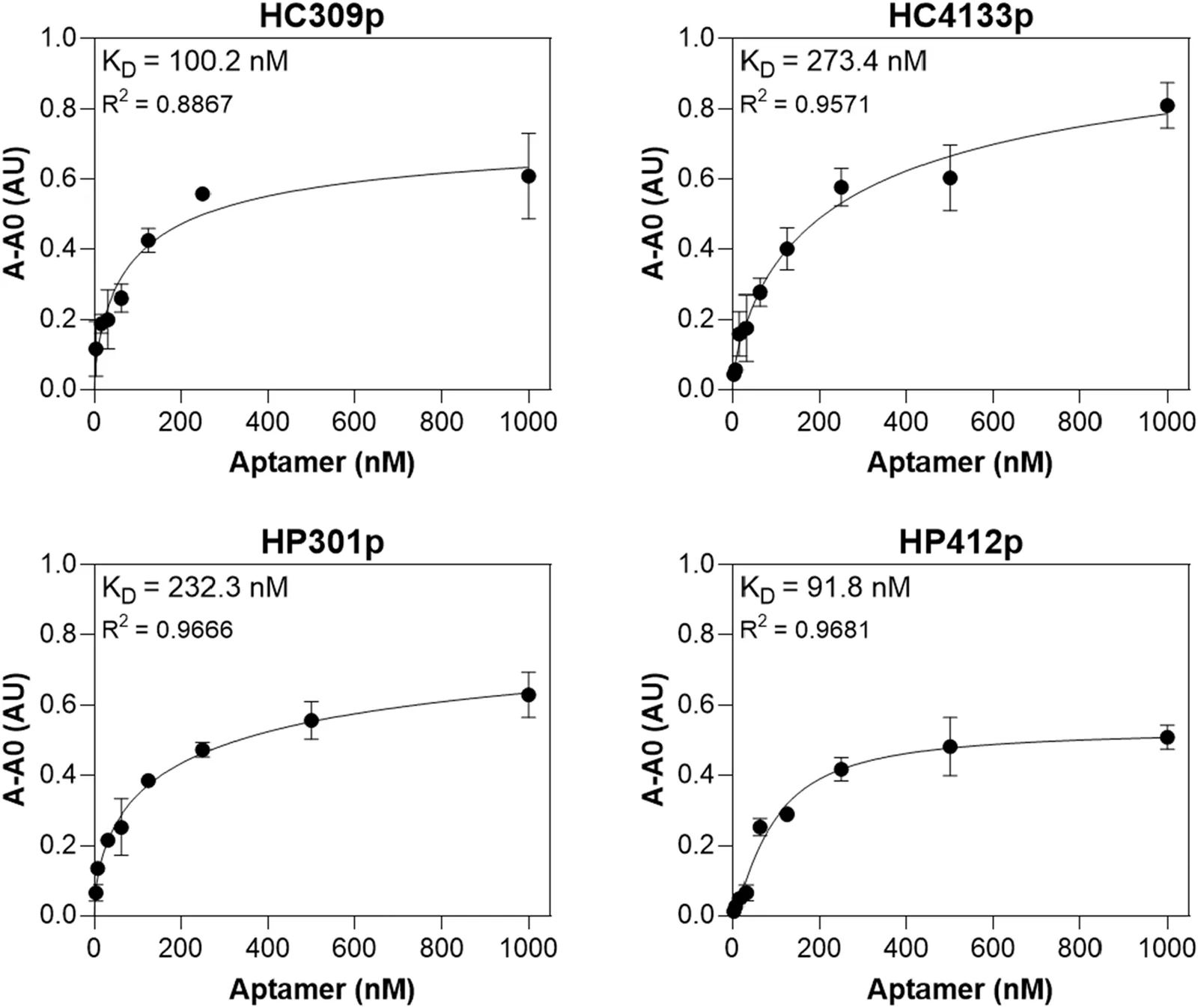
Determination of the affinity dissociation constants (*K*_D_ values) of the selected aptamers by indirect ELAA. Each biotinylated aptamer was titrated in duplicate against plate-immobilized *M. tuberculosis* ESAT-6/CFP-10.

In BLI, the K_D_ values calculated were in the higher nanomolar range as shown in Figure 7, 424 and 356 nM for aptamers HC309p and HC4133p, respectively from capture-SELEX and 396 and 348 nM for HP301p and HP412p, respectively, deriving from bait-SELEX. Similar kinetic profiles were observed for all aptamers, with the association rates (*k*_on_) in the range of 1.0–1.2 × 10^5^ M^–1^ s^–1^ and the dissociation rates (*k*_off_) at 4.0–4.7 × 10^–4^ s^–1^. The low *k*_off_ values indicate the formation of stable complexes, which may enhance the performance of bioassays by limiting losses, especially during washing steps. The binding affinities determined by BLI were slightly higher than those obtained by ELAA, which may result from steric hindrance and restricted conformational flexibility of aptamers tethered onto the sensors. Immobilization can limit the ability of the aptamers to undergo optimal conformational switching when they have interacted with the heterodimer target in the solution. Nevertheless, the affinities of the aptamers selected by both bait-SELEX and capture-SELEX, as determined by ELAA and BLI, remain within the same range regardless of whether the target or the aptamer were immobilized.

**Figure 7:**
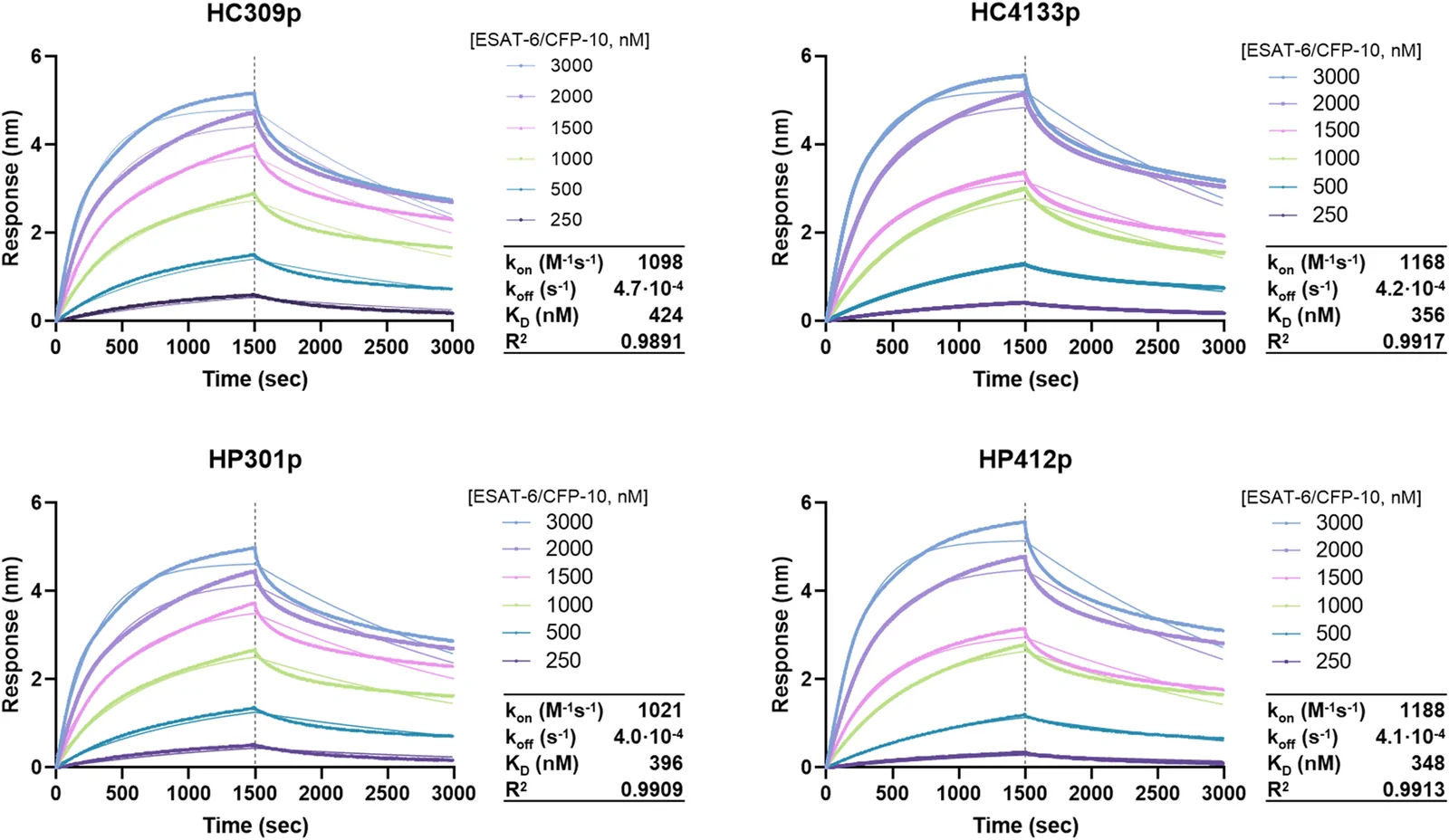
Determination of the binding kinetics of the selected aptamers by Biolayer Interferometry (BLI). Each biotinylated aptamer was immobilized on streptavidin biosensors and its binding kinetics to serially diluted *M. tuberculosis* ESAT-6/CFP-10 heterodimer was monitored during 25 min of association and 25 min of dissociation.

As commented previously, only two selections have been reported to date against the *M. tuberculosis* ESAT-6/CFP-10 heterodimer (Table S4). The first one employed filter membrane SELEX, where the library was incubated with the target in solution and a nylon membrane was used for partitioning.^57^ Screening of aptamer candidates CSIR 2.2, 2.11, 2.15, 2.19, and 2.21 identified after six rounds was carried out by ELAA, and it revealed that while they all recognized the heterodimer, they only bound to CFP-10 and no interaction was observed with ESAT-6. Their K_D_ values for CFP-10 were found to be within the low-nanomolar range (1.6–21.5 nM) by surface plasmon resonance (SPR).^57^ Since these values were determined for one subunit only and not the entire heterodimer, the binding properties of these aptamers cannot be directly compared to the ones reported in this work. Monomer-based measurements reflect only partial target architecture and may misrepresent the binding strength for the native heterodimeric target. The other report targeted a single-chain ESAT-6.CFP-10 (CE) protein variant which was produced recombinantly by fusing the C-terminal of CFP-10 to the N-terminal of ESAT-6.^48^ A microplate-SELEX with the CE protein immobilized on microplate wells was carried out for 17 rounds and the binding affinities of the identified aptamers CE15 and CE24 were in the high nanomolar range (160–375 nM) as determined by ELAA. The affinity of these aptamers is comparable to that of the aptamers reported in this work and evaluated with an equivalent plate-based ELAA format, even though the CE target variant differs from the “native-like” heterodimer formed *in vitro* and used in this work. Overall, the library-immobilized selection strategies reported here yielded aptamers with moderate binding affinities, with higher *K*_D_ values being determined with BLI (356–424 nM) compared to indirect ELAA (92–273 nM), with these differences attributable to assay-specific limiting factors. In ELAA, the random adsorption of the heterodimer on the well surface may expose epitopes preferentially recognized by the aptamers or promote partial target multimerization, enhancing apparent avidity and leading to lower apparent *K*_D_ values. In BLI, the oriented 5′-biotin/streptavidin immobilization and enforced lateral spacing between aptamers on the sensors may impose steric constraints that reduce their flexibility, contributing to higher observed *K*_D_ values as compared to the solution-phase ELAA. Despite these differences, the selected aptamers, especially those from bait-SELEX, remain promising candidates for assay development, as they bind the “native-like” heterodimer with good affinity.

### Binding Activity of the Selected Aptamers in Biological Matrices

Aptamers for biosensors and diagnostic assays can offer stability, specificity, and ability to function in complex biological matrices, particularly nonsputum samples such as urine, to enable TB detection applications.^44,70,71^ To assess matrix compatibility, the binding of the aptamers to the ESAT-6/CFP-10 heterodimer was assessed in binding buffer spiked with human urine at different dilutions. As can be observed in Figure S7, urine elicited no significant binding loss across dilutions, a pattern consistent with all four candidates from both SELEX variants, confirming the suitability of the aptamers for urine-based TB diagnostics.

### *M. tuberculosis* ESAT-6/CFP-10 Antigen Detection with a Hybrid Antibody–Aptamer Sandwich Assay

Hybrid enzyme-linked antibody–aptamer sandwich assays (ELAASAs) combine the advantages of both antibodies and aptamers in biosensing and detection assays. With the aim of developing such an assay for *M. tuberculosis* targeting the ESAT-6/CFP-10 heterodimeric antigen, as previously described, the bait-SELEX strategy was designed in this work to direct the SELEX toward the selection of aptamers that can form a sandwich complex with a CFP-10-binding antibody and the target. As polyclonal antibodies recognize multiple epitopes on a target, it was considered the most suitable choice in this case, as it would maximize the number of accessible binding regions on the antigen and reduce the possibility of selecting aptamers that bind epitopes overlapping with the antibody. As stated earlier, an initial screening was carried out to identify bait-SELEX-deriving aptamers providing the highest sensitivity in the detection of the heterodimer. This screening showed that HP301p and HP412p aptamers were the most promising candidates (Figure 5c); therefore, they were chosen for further characterization. To maximize the performance of the ELAASAs employing either aptamer for detection, the concentrations of both antibody and aptamers were optimized through checkerboard titrations. The capture antibody was used at 1.25–20 μg·mL^–1^, while the detection biotinylated aptamer was employed in the range of 125–4000 nM. As can be seen in Figure S8, saturating signals were obtained for ≥10 μg·mL^–1^ of antibody, while the highest signals were for 1–2 μM of aptamers. For the final assay, 1 μM of reporter aptamers was used since 2 μM resulted in higher background signals in the absence of the analyte. Calibration curves were finally constructed (10 μg·mL^–1^ of capture anti-CFP-10 antibody and 1 μM of each biotinylated reporter aptamer) with the concentration of the target heterodimer ranging from 312 nM to 20 μM. The limit of detection (LOD) of the ELAASAs were 188 nM (4.3 ng·μL^–1^) and 1178 nM (26.7 ng·μL^–1^) when HP301p and HP412p aptamers were used as reporters, respectively (Figure 8).

**Figure 8:**
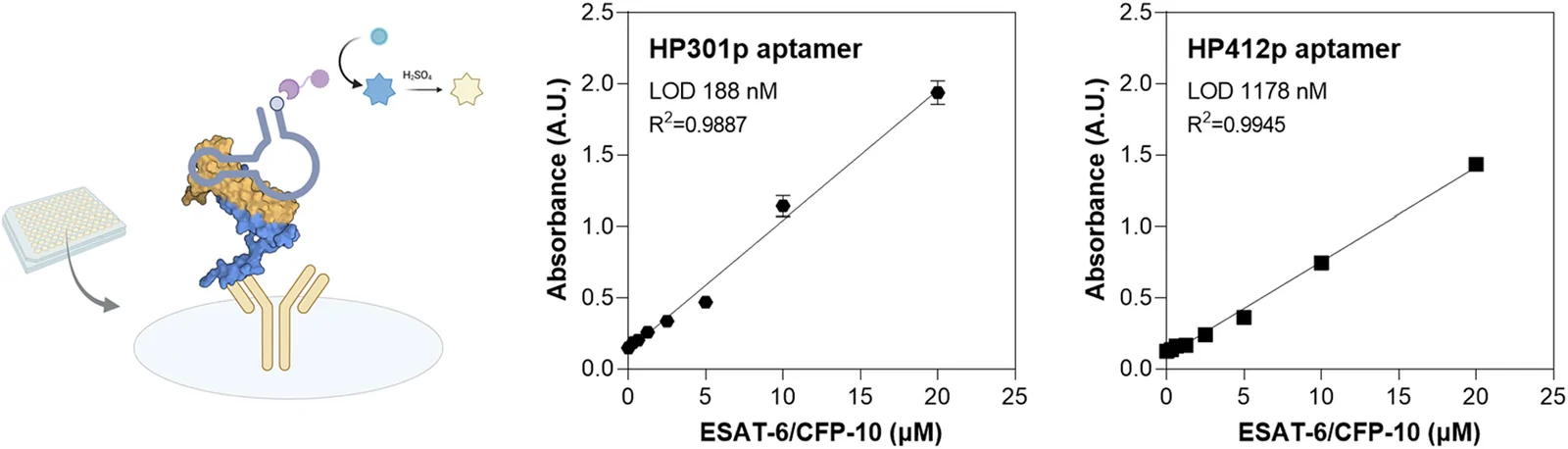
Detection of *M. tuberculosis* ESAT-6/CFP-10 heterodimer with hybrid antibody–aptamer assays. *n* = 3, MV ± SD.

Few assays have been developed to date using the aptamers previously selected against the “native-like” or single-chain ESAT-6/CFP-10 heterodimer (Table S4). Rotherham et al.^57^ successfully employed one of the selected aptamers (CSIR 2.11) in an indirect ELAA for detecting antigens in active TB sputum samples on microplate wells, but the sensitivity of the assay was not explicitly assessed. The same aptamer was also utilized by Azmi et al.^100^ to develop an electrochemical aptasensor with an LOD of 1.52 ng·mL^–1^. The CE15 and CE24 aptamers selected by Tang et al.^58^ against the single-chain CE protein were employed in hybrid sandwich assays on microplates in combination with a polyclonal CE-specific antibody for the detection of TB in serum from active PTB patients, but the analytical sensitivity of those assays was not determined. The same aptamers were employed in a sandwich format for the development of a nanocomposite dual-signal electrochemical aptasensor which exhibited an LOD of 2.88 fg·mL^–1^ for ESAT-6 alone.^72^ While the sensitivity of the aptasensor for detecting the heterodimer was not studied, it successfully detected TB in patient serum samples.

Overall, although the LOD of the proof-of-concept plate-based hybrid sandwich assay reported here is modest, the integration of the antibody/aptamer pair into a more sensitive assay platform is a potential path forward. This would leverage antibody sensitivity with the aptamer specificity, stability, and versatility for the development of cost-effective TB antigen detection suited for early TB detection in field settings.

## Conclusions

This work evaluated immobilized-library strategies for aptamer selection targeting the *M. tuberculosis* ESAT-6/CFP-10 heterodimer, a key biomarker secreted during active infection. Two DNA libraries with distinct architectures were compared: the complex CS3, with dual random regions flanking a constant hybridization domain for magnetic bead immobilization, and the simpler CS4, featuring a single shorter random region tethered through primer site hybridization. These libraries facilitated two SELEX modalities, a conventional capture-SELEX yielding target-displacing aptamers and a novel bait-SELEX using enriched target-elution pools from capture-SELEX for incubation with target-specific antibody-coated beads for the isolation of aptamers forming aptamer/target/antibody sandwiches. Next-generation sequencing analysis revealed superior enrichment in capture-SELEX with the CS3 library compared to CS4, while bait-SELEX achieved similar enrichment in fewer rounds (six versus 11 for capture-SELEX), regardless of the library used, suggesting that the additional antibody–bait step effectively focused the selection on sandwich-compatible binders regardless of library architecture. Selected aptamer candidates displayed comparable apparent affinity dissociation constants when measured by enzyme-linked aptamer assay (ELAA) with immobilized target (*K*_D_ of 92–273 nM) and biolayer interferometry (BLI) with immobilized aptamers (348–424 nM). These results are consistent with the reduced binding efficiency of immobilized aptamers, potentially resulting from conformational constraints and steric effects. Matrix tolerance experiments showed that aptamer binding was not affected in diluted urine, showing compatibility to noninvasive and nonsputum specimens.

An alternative strategy for selecting aptamers capable of forming sandwiches with an antibody was previously reported by Kang et al.^73^ This heterogeneous sandwich SELEX process was based on the immobilization of a target-specific antibody on microplate wells for capturing the target and the interrogation of an ssDNA library in solution. With this strategy, they selected aptamers against influenza A nucleoprotein after ten rounds with affinity dissociation constants in the nanomolar range (84.5–125.7 nM) which, when applied to a heterogeneous sandwich assay with the antibody, achieved an LOD of 1.02 ng·mL^–1^. The performance of those aptamers in terms of binding affinity and assay sensitivity is comparable to the one reported herein after employing the bait-SELEX strategy which was completed in fewer selection rounds.

Finally, aptamers isolated by bait-SELEX were successfully employed in hybrid antibody/aptamer sandwich assays, yielding limits of detection of 188 nM for the CS3-derived aptamer HP301p and 1.18 μM for the CS4-derived one HP412p. Overall, the results demonstrate that immobilized-library selection strategies can successfully generate functional aptamers against the *M. tuberculosis* ESAT-6/CFP-10 heterodimeric antigen, and that the bait-SELEX approach provides a practical and rapid route to directly enrich aptamers compatible with antibody-based sandwich assay formats. By comparing the two selection modalities employing libraries with different architectures, it can also be concluded that aptamer selection is mainly target-driven and that selection conditions can influence the speed and functionality of selected aptamers. Taken together, these findings provide a foundation for further optimizations of the selected aptamers and support the future integration and development as components toward next-generation TB antigen detection tools, especially with ending TB in 2030 is one of the core targets of Sustainable Development Goals (SGDs) in 2026.

## Materials and Methods

### ESAT-6/CFP-10 Heterodimeric Complex Formation

ESAT-6 and CFP-10 were expressed from their respective plasmids in *E. coli* and purified by immobilized metal affinity chromatography (IMAC) as detailed in the Supporting Information (Figure S1). Purified proteins were incubated at 1:1 equimolar ratio in neutral pH buffer (PBS pH 7.4) for 2 h at room temperature (22–25 °C) as described previously.^51,53^ Complex formation was confirmed by native PAGE on ice for 3–4 h at 50 V. Intrinsic tryptophan fluorescence spectra of ESAT-6, CFP-10, ESAT-6/CFP-10 heterodimer, as well as combined ESAT-6 and CFP-10 without prior incubation at 5 μM each in PBS (pH 7.4) were acquired on a Cary Eclipse Fluorescence Spectrometer (Agilent). The excitation wavelength was set at 295 nm and fluorescence emission at 310–415 nm was recorded at a scan rate of 120 nm/min in 1 nm interval. The final spectra were corrected by subtracting buffer background signals.

### Capture-SELEX and Bait-SELEX Library-Immobilized Aptamer Selection Strategies

A capture-SELEX and a two-phase bait-SELEX approaches were employed to select aptamers for the *M. tuberculosis* ESAT-6/CFP-10 heterodimer using two synthetic single-stranded DNA (ssDNA) libraries (CS3 and CS4) in parallel as the starting pools. Each ssDNA library was hybridized with its respective biotinylated capture probe (Table S1) and immobilized on streptavidin-coated magnetic beads (SA-MB) as follows: the library was mixed with its capture probe at 1:1 molar ratio in binding buffer (PBS with 1.5 mM MgCl_2_), heated for 95 °C for 5 min, and gradually cooled down to room temperature (22–25 °C). The mixture was incubated for 2 h at room temperature for hybridization under gentle rotation, followed by overnight incubation with SA-MB previously washed three times in binding and washing buffer (10 mM Tris-HCl pH 7.5, 2 M NaCl, 1 mM EDTA). Following magnetic separation and washing, capture probe-hybridized ssDNA pool (CP-ssDNA) was retained on the beads for selection.

For capture-SELEX, the immobilized ssDNA libraries were first incubated with binding buffer at 28 °C for 15 min at 700 rpm to remove weakly bound ssDNA from the CP-ssDNA on SA-MB (pre-elution). The eluted ssDNA was discarded and the immobilized libraries on the magnetic beads were resuspended with binding buffer and incubated at room temperature for 1–2 h under rotation (background elution). After removal of the supernatant with eluted sequences, a positive selection step was performed by incubating the immobilized libraries with ESAT-6/CFP-10 heterodimer in binding buffer for 1–2 h at room temperature under rotation (target elution). Eluted ssDNA complexed with the target from the positive selection step was used to prepare ssDNA for further selection rounds. A total of 11 rounds of capture-SELEX were carried out.

For bait-SELEX, the ssDNA pools generated after four rounds of capture-SELEX were used for another round of capture-SELEX as described above, followed by the antibody-assisted isolation of target-bound sequences. Specifically, the collected target-elution fractions from capture-SELEX were incubated with an anti-CFP-10 IgG antibody immobilized on magnetic beads for 1 h at room temperature under rotation to capture target-bound sequences able to form sandwich complexes with the antibody. Following magnetic separation, the beads were washed with PBS, and bound ssDNA was eluted with 0.1 M glycine, pH 2.5, followed by neutralization with 1 M Tris-HCl, pH 8.8. The recovered ssDNA was used to generate ssDNA for the following rounds of bait-SELEX, consisting of consecutive capture-SELEX and antibody-assisted steps. A total of six rounds of bait-SELEX were carried out. The preparation of the antibody–magnetic beads conjugate is detailed in the Supporting Information and depicted in Figure S2.

The stringency of both selection strategies was increased by gradually decreasing the concentration of the target ESAT-6/CFP-10 heterodimer as well as the duration of its incubation with the immobilized libraries. The conditions used during the selections are summarized in Table S2.

### Monitoring the Evolution of the Selections

The progress of the selections was monitored by quantitative PCR (qPCR) and melt curve analysis as previously described^65,74^ with minor modifications. The amount of ssDNA in the eluted fractions of target-ssDNA complexes was first quantified by qPCR. A total volume of 10 μL reactions contained 1x PowerUP SYBR Green Master Mix, 200 nM each of forward and reverse library-specific primers, and 1 μL of eluted target–ssDNA complexes diluted 10-fold as template. Amplification was performed on a StepOne Real-Time PCR System (Applied Biosystems, USA) with the following thermal cycling conditions: 2 min at 50 °C (UDG activation), 2 min at 95 °C (Dual-Lock DNA polymerase activation), followed by 40 cycles of 15 s at 95 °C, 15 s at 52 °C, and 15 s at 72 °C. All amplification reactions were performed in duplicate with nontemplate controls (NTC) included in each run. Quantification was performed using a seven-point calibration curve of 10-fold serial dilution of the standard spanning a concentration range of 100 – 0.0001 ng·μL^–1^ prepared from the starting (unselected) ssDNA library. Enrichment was expressed as the ratio of the calculated amount of eluted ssDNA (target-bound aptamers) divided by the starting amount of ssDNA used in each selection round. For melt curve analysis, the PCR reactions from the previous step were heated from 60 to 95 °C in 0.5 °C/min increments, with continuous monitoring of SYBR Green fluorescence. Melting profiles and derivative peaks (−dF/dT) were generated using StepOne software (Applied Biosystems, USA) to evaluate amplification and monitor ssDNA enrichment across selection rounds.

### Next-Generation Sequencing and Bioinformatics Analyses

Fractions containing target-bound ssDNA from several selection rounds as well as the initial libraries were PCR-amplified with barcoded library-specific primers under the cycling conditions described earlier. The number of PCR cycles was optimized to avoid overamplification. Barcoded PCR products were then purified using the DNA Clean and Concentrator-5 kit (Zymo Research, USA) according to the manufacturer’s protocol. Purified pools were subjected to high-throughput sequencing on an Ion Torrent Ion GeneStudio S5 system (Thermo Fisher Scientific, USA). Raw sequencing reads were processed using the Galaxy software (https://usegalaxy.org/). The length of the reads was limited to ±5 bp of the length of each library (usable reads), and finally, they were collapsed into unique sequences. The enrichment was calculated as the percentage of sequences with more than one copy within each selection round analyzed. The 100 most abundant sequences were subjected to multiple sequence alignment using Clustal Omega (https://www.ebi.ac.uk/jdispatcher/msa/clustalo) and clustered to identify conserved families. MEME suite (http://meme-suite.org) was used to screen for conserved sequence motifs within the 100 most abundant sequences. Secondary structure predictions and corresponding free energy (Δ*G*) values were predicted using the Mfold web server^75^ (https://www.unafold.org/mfold/applications/dna-folding-form.php) and visualized using RNAcanvas.^76^ Candidate aptamers were selected based on the enrichment and predicted structural stability for downstream validation.

### Enzyme-Linked Aptamer Assay (ELAA)

Indirect ELAA was carried out to evaluate the binding properties of the selected aptamers. Nunc MaxiSorp 96-microplate wells were coated with 50 μL of 10 μM ESAT-6/CFP-10 heterodimer in 50 mM carbonate–bicarbonate buffer, pH 9.6 overnight at 4 °C. The wells were blocked with 200 μL of 5% (w/v) skimmed milk in PBS containing 0.05% (v/v) Tween-20 (PBST) for 1 h, followed by the addition of biotinylated aptamers which were previously refolded by heating at 95 °C for 5 min and gradually cooled down to 25 °C. For screening of the aptamer candidates, each aptamer was used at 500 nM in binding buffer, whereas different aptamer concentrations (3.9–1000 nM) were used for the determination of the affinity dissociation constants (K_D_ values). After 1 h incubation, 50 μL of SA-pHRP (50 ng·mL^–1^ in PBST) were added and incubated for 30 min, followed by a 15 min incubation with 50 μL of 3,3′,5,5′-tetramethylbenzidine (TMB) substrate and termination of color development by the addition of 50 μL of 1 M H_2_SO_4_. All incubation steps were carried out at room temperature (22–25 °C) under mild agitation unless otherwise stated. The wells were washed with PBST (3 × 200 μL) after each step, and all samples were assayed in duplicate. Absorbance was measured at 450 nm using the SpectraMax M2 microplate reader (Molecular Devices). To calculate the affinity dissociation constants (*K*_D_) of the aptamers, background-corrected absorbance values were plotted against aptamer concentration, and the data were fitted to the model “Specific binding with Hill slope” in GraphPad Prism 10 (GraphPad Software, USA). To test the compatibility of the aptamers with biological fluids, each aptamer (1000 nM) was used to bind plate-immobilized ESAT-6/CFP-10 heterodimer as described above in human urine diluted 1:10, 1:50, or 1:100 in binding buffer. The signals obtained in each urine dilution were normalized to the signals generated in the absence of it (binding buffer alone) to calculate % relative binding.

### Biolayer Interferometry (BLI)

BLI analyses were carried out on an Octet RED96e (ForteBio) instrument using streptavidin (SA) biosensors at 25 °C. SA biosensors were hydrated in the sensor tray in milli-Q water for at least 10 min, equilibrated in binding buffer and then added to a 1000 nM solution of 5́-biotinylated aptamer in binding buffer for 5 min. The aptamer-loaded sensors were blocked with assay buffer (0.6 M sucrose and 0.05% (v/v) Tween-20 in binding buffer) and subsequently immersed into wells containing varying concentrations of the target protein (250 to 3000 nM) in the same buffer for kinetic measurements. Each step was performed with a shaking speed of 1000 rpm and with a baseline step of 300 s. Association was monitored for 1500 s, followed by transfer into wells containing only binding buffer to monitor dissociation for an equal duration. A reference sensor without loaded aptamer was included in each run to correct for nonspecific signal drift. Data were reference-subtracted and fitted to a 1:1 model using “Association then Dissociation” binding kinetics in GraphPad Prism 10 software to calculate the association (*k*_on_) and dissociation (*k*_off_) rates and the *K*_D_ values.

### Hybrid Antibody–Aptamer Assay for *M. tuberculosis* ESAT-6/CFP-10 Heterodimer Detection

Aptamers isolated from bait-SELEX were screened in antibody–aptamer sandwich assays for selecting the candidate best suited for a sandwich assay. The anti-CFP-10 polyclonal IgG (50 μL of 5 μg·mL^–1^ in 50 mM carbonate–bicarbonate buffer pH 9.6) was used to coat Nunc MaxiSorp 96-microplate wells overnight at 4 °C, followed by blocking with 200 μL of 5% (w/v) skimmed milk in PBST for 1 h at room temperature under mild agitation. The ESAT-6/CFP-10 heterodimer (50 μL of 20 μM in PBS) was then added and incubated for 1 h at room temperature, followed by the addition of refolded biotinylated aptamers (50 μL of 500 nM in binding buffer). Following another 1 h of incubation and washing, SA-pHRP (50 μL of 50 ng·mL^–1^ in PBST) was added for 30 min, the wells were washed again, and the TMB substrate (50 μL) was added for 15 min before stopping the enzymatic reaction with 1 M H_2_SO_4_ (50 μL). For assay development, the concentrations of anti-CFP-10 IgG antibody (capture) and biotinylated aptamer (detection) were optimized by checkerboard titrations, using the IgG at 1.25–20 μg·mL^–1^ and the biotinylated aptamers at 125–4000 nM. Finally, calibration curves were constructed under optimized capture and detection probes concentrations using the ESAT-6/CFP-10 heterodimer target at 0–20 μM. The limit of detection (LOD) was determined from linear calibration curves plotted in GraphPad Prism 10 as the blank plus three times its standard deviation.

## Supplementary Material



## Notes

Materials; expression and purification of *M. tuberculosis* ESAT-6 and CFP-10 antigens (Figure S1); immobilization of anti-CFP-10 IgG antibody on magnetic beads; functionalization of anti-CFP-10 antibody on carboxylic acid-activated magnetic beads (Figure S2); oligonucleotide libraries, docking probes, and primer sequences used in this study (Table S1); PCR amplification and ssDNA generation; conditions used during Capture-SELEX and Bait-SELEX in parallel selections with CS3 and CS4 libraries (Table S2); progress of the capture-SELEX selections monitored by qPCR (Figure S3); progress of the bait-SELEX selections monitored by qPCR (Figure S4); selected aptamer candidates (Table S3); predicted secondary structures of aptamer candidates from capture-SELEX (Figure S5); predicted secondary structures of aptamer candidates from bait-SELEX (Figure S6); interactions of the aptamers with ESAT-6/CFP-10 heterodimer in binding buffer spiked with artificial human urine at different dilutions (Figure S7); optimization of the concentrations of the α-CFP-10 IgG antibody (capture) and biotinylated aptamer (detection) in hybrid antibody–aptamer sandwich assay (Figure S8); and aptamers selected against the *M. tuberculosis* ESAT-6/CFP-10 heterodimeric antigen to date (Table S4) (PDF)
