# Supplementary material for: Advancing Tuberculosis Antigen Detection by Selecting Sandwich-Compatible Aptamers Against the Mycobacterium tuberculosis ESAT-6/CFP-10 Heterodimer Using Library-Immobilized Strategies
Source: ACS Infect Dis. 2026 Aug 31;12(10):3472–86. doi: 10.1021/acsinfecdis.6c00529 (PMC13660414; doi:10.1021/acsinfecdis.6c00529)
Supplement: Supplementary file 1 [file id6c00529_si_001.pdf]

SUPPORTING INFORMATION

Advancing tuberculosis antigen detection by selecting sandwich-compatible aptamers against the *Mycobacterium tuberculosis* ESAT-6/CFP-10 heterodimer using library-immobilized strategies

Shaira Jane Acosta, [†] *M. Carmen Bermudo Redondo,* [†] *Lucie Mužíková Čechová,*[‡] *Marek Ondruš,* [‡] *Vasso Skouridou,* [†]* *& Ciara K. O'Sullivan,* [†,§]*

[†] Interfibio Consolidated Research Group, Departament d'Enginyeria Química, Universitat Rovira i Virgili, 43007 Tarragona, Spain

[‡] Institute of Organic Chemistry and Biochemistry, Czech Academy of Sciences, Flemingovo nam. 542/2, 160 00 Prague 6, Czech Republic

[§] Institució Catalana de Recerca i Estudis Avancats (ICREA), 08010 Barcelona, Spain

*Corresponding authors: ciara.o.sullivan@urv.cat, vasoula,skouridou@urv.cat

Materials

All chemicals and buffers were prepared using molecular- and analytical-grade reagents purchased from Fisher Scientific (Spain) or Merck (Spain) in milli-Q water. Plasmids pMRLB.7 and pMRLB.46 containing genes Rv3875 for ESAT-6 and Rv3874 for CFP-10, respectively from *Mycobacterium tuberculosis* were obtained from BEI Resources (NIAID, NIH, USA). Phosphate-buffered saline (PBS; 10 mM phosphate, 137 mM NaCl, 2.7 mM KCl, pH 7.4), BupH™ carbonate-bicarbonate buffer, *Escherichia coli* BL21(DE3) pLysS cells, HisPur™ Cobalt Resin, DreamTaq DNA polymerase and DreamTaq buffer, dNTPs, PowerUp™ SYBR Green master mix, molecular-biology grade agarose, GeneRuler Low Range DNA ladder, streptavidin-coated magnetic beads (Dynabeads™ M-270 Streptavidin), carboxylic acid-activated magnetic beads (Dynabeads™ M-270 Carboxylic Acid), neutravidin-coated plates, Luria Bertani (LB) growth medium and LB agar, ampicillin, chloramphenicol, isopropyl-D galactopyranoside (IPTG) and PageBlue protein staining solution were obtained from Fisher Scientific (Spain). PD-10 desalting columns were from Cytiva Life Sciences (Spain). *M. tuberculosis* anti-CFP10 antibody produced in rabbit, synthetic human urine (Surine™ Negative Urine Control) and skimmed milk powder were from Merck (Spain). Biotium GelRed® nucleic acid gel stain was from Biogen Cientifica (Spain), 3,3′,5,5′-tetramethylbenzidine (TMB) from Surmodics (USA) and streptavidin-polyHRP80 (SA-pHRP) from SDT (USA). Pre-cast 4-15% polyacrylamide gels for SDS-PAGE and native PAGE were purchased from Bio-rad (Spain). Zymo Research spin-column-based DNA purification kits were provided by Ecogen (Spain) and Octet® Streptavidin (SA) Biosensors from Sartorius. All oligonucleotides were synthesized by Biomers.net (Germany) and were reconstituted with nuclease-free water.

Expression and purification of *M. tuberculosis* ESAT-6 and CFP-10 antigens

The expression vectors pMRLB.7 and pMRL.46 encoding for His_6-tagged ESAT-6 and CFP-10, respectively, were individually transformed in chemically competent *E. coli* BL21(DE3) pLysS cells following the manufacturer's instructions. Transformed cells were grown at 37°C and 180 rpm in Luria Bertani medium supplemented with 150 µg/mL ampicillin and 34 µg/mL chloramphenicol until mid-log phase (optical density OD600 ~ 0.6). Protein expression was induced using 0.2 mM isopropyl-D galactopyranoside (IPTG) and the cultures were grown for a further 4 h at 30°C and 180 rpm. Cells were harvested by centrifugation and resuspended in protein extraction buffer (PBS pH 7.4 with 0.3 M NaCl, 0.3 mg/mL lysozyme, 5% (v/v) glycerol, 1% (v/v) Triton X-100, 10 µg/mL each of DNAseI and RNAseA, 1 mM DTT, 1 mM phenylmethylsulfonyl fluoride and 5 mM imidazole). The cells were lysed for 10 min on ice by sonication (5 sec on, 5 sec off, amplitude 60%). The cell lysates were cleared via centrifugation at 10,000 rpm for 10 min at 6°C and the soluble fractions were filtered using 0.22 µm PES filters. The recombinant His-tagged proteins were purified by immobilized metal affinity chromatography (IMAC). Briefly, HisPur™ Cobalt resin was loaded to a gravity-flow column and pre-equilibrated with equilibration buffer (PBS pH 7.4 with 0.3 M NaCl) with 5 mM imidazole. The cleared soluble cell lysates were added to the columns and incubated for 30 min under gentle rotation at 22ºC. After removal of the unbound fractions, the resins were washed with 10 mM and 20 mM of imidazole in equilibration buffer prior to elution of resin-bound proteins with 200 mM imidazole in the same buffer. The process was monitored by sodium dodecyl-sulfate polyacrylamide gel electrophoresis (SDS-PAGE) and the gels were stained with PageBlue protein staining solution. Protein fractions with high purity (>90%) were pooled, concentrated using PES spin concentrators and finally the buffer

was exchanged to storage buffer (PBS, pH 7.4) using PD-10 desalting columns. Proteins were quantified by ultraviolet spectroscopy (A280 nm) using molar extinction coefficients predicted by Expasy ProtParam tool (https://web.expasy.org/protparam/) based on the expected amino acid sequences of the expressed proteins. Expressed proteins were assessed using indirect enzyme-linked immunosorbent assay (ELISA) to confirm their immunogenic response to commercial antibodies.

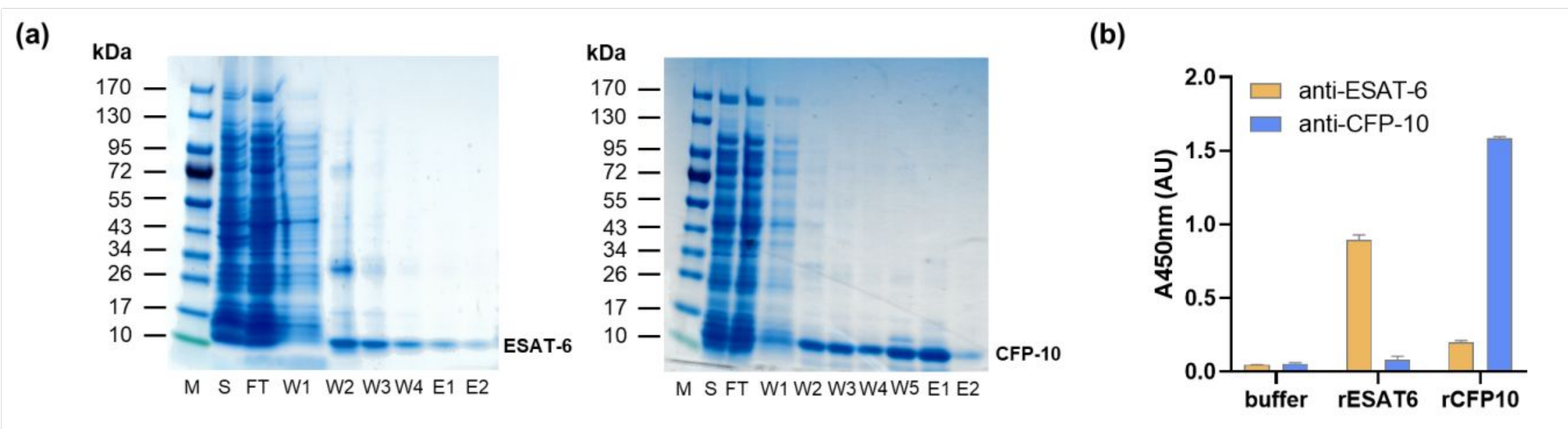

Figure S1. Expression and purification of *M. tuberculosis* ESAT-6 and CFP-10 antigens. (a) SDS-PAGE analysis confirming the molecular weight of respective proteins and (b) indirect ELISA showing antibody-based detection of the purified proteins.

Immobilization of anti-CFP-10 IgG antibody on magnetic beads

Covalent coupling of anti-CFP-10 rabbit polyclonal (pAb) IgG antibody to carboxylic magnetic beads was performed using carbodiimide chemistry. In brief, carboxylic magnetic beads (Dynabeads™ M-270 Carboxylic Acid) were washed three times with 25 mM MES buffer [2-(N-morpholino)ethanesulfonic acid] pH 5.0. Carboxyl groups were activated by incubation with 100 μL of freshly prepared solution of 50 mg/mL each 1-ethyl-3-(3-dimethylaminopropyl)

carbodiimide (EDC) and N-hydroxysuccinimide (NHS) in MES buffer for 30 min at 22ºC with gentle mixing. The activated beads were washed with MES and incubated with 50 µg of anti-CFP-10 pAb for 30 min at 22ºC. After washing three times with PBS, the magnetic beads (anti-CFP-10@MB-COOH) were resuspended in 50 µL PBS. To verify antibody immobilization, bead-based enzyme-linked immunosorbent assay (ELISA) was performed (Figure S2). Anti-CFP-10@MB-COOH beads and control beads prepared in parallel without the antibody were transferred to microtubes and washed 3x with PBS containing 0.05% v/v Tween-20 (PBST). The beads were incubated with horseradish peroxidase-conjugated anti-rabbit IgG at 1:10,000 dilution in PBST for 30 min at room temperature under gentle rotation. The beads were washed with PBST, incubated with TMB substrate until color developed and the reaction was terminated with 1 M H_2SO_4. The supernatants were collected by magnetic separation and the absorbance was measured at 450 nm using a microplate reader (Agilent, USA).

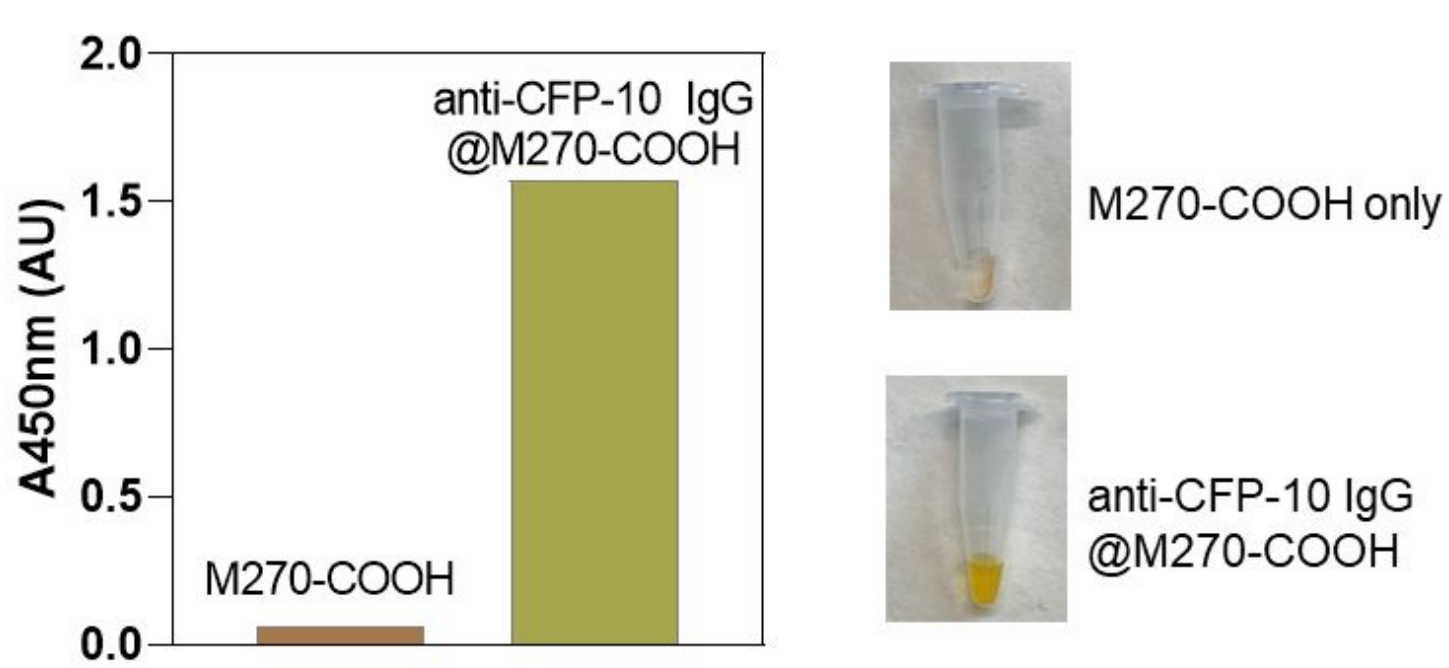

Figure S2. Functionalization of anti-CFP-10 antibody on carboxylic acid-activated magnetic beads verified using bead-based enzyme-linked immunosorbent assay (bead-ELISA).

PCR amplification and ssDNA generation

PCR amplification was performed in 100 µL reactions containing 1x DreamTaq buffer, 200 µM dNTPs, 200 nM forward primer, 200 nM 5’-phosphorylated reverse primer, 1 U DreamTaq DNA polymerase, and recovered ssDNA from each elution fraction (diluted 1:10) as the template. Thermal cycling was carried out using a Bio-Rad T100 thermocycler with an initial denaturation of 95ºC for 2 min, followed by 5-25 cycles of 95ºC for 15 sec, 52ºC for 15 sec, and 72ºC for 15 sec, with a final extension 72ºC for 5 min. PCR products were resolved on 2.6% agarose/TBE gels containing GelRed nucleic acid stain. Electrophoresis was performed at 120 V for 30 min and gels were imaged using a UV transilluminator (Analytik Jena). The optimal number of PCR cycles required for DNA amplification after each selection round was determined empirically to prevent over-amplification and minimize PCR by-products. Double-stranded DNA PCR products were converted to ssDNA by lambda exonuclease digestion at 37ºC for 1.5 h, followed by spin-column-based ssDNA purification. The concentration of purified ssDNA was determined spectrophotometrically at 280 nm using SimpliNano™ (GE Healthcare Technologies).

Table S1. Oligonucleotide libraries, docking probes, and primer sequences used in this study.

Library/primer/probe	Sequence
CS3 library	5'-AAGCATCCGCTGGTTGAC-(N10)-TGAGGCTCGATC-(N40)-ATGCCATTTGGGCTGCTC-3'
CS3 capture probe	Biotin-TEG-CTG-HEGL-GATCGAGCCTCA
CS3 Fw primer	5'-AAGCATCCGCTGGTTGAC-3'
CS3 Rv primer	5'-GAGCAGCCCAAATGGCAT-3'
CS4 library	5'-AGCAGTCGTGGCGTAATA-(N30)-GATCTTGGACCCTGCGAA-3'
CS4 capture probe	5'-Biotin-TEG-CTG-HEGL-TTCGCAGGGTC-3'
CS4 Fw primer	5'-AGCAGTCGTGGCGTAATA-3'
CS4 Rv primer	5'-TTCGCAGGGTCCAAGATC-3'

Table S2. Conditions used during Capture-SELEX and Bait-SELEX in parallel selections with CS3 and CS4 libraries.

Selection strategy	Round	Library	Library	ESAT-6/CFP-10 heterodimer	Incubation time
Capture-SELEX	1	CS3 or CS4	200 pmol	20 µM	2 h
	2		15 pmol	20 µM	2 h
	3		5 pmol	20 µM	2 h
	4		5 pmol	20 µM	1 h
	5		15 pmol	20 µM	1 h
	6		15 pmol	20 µM	1 h
	7		15 pmol	20 µM	1 h
	8		15 pmol	10 µM	1 h
	9		15 pmol	10 µM	1 h
	10		15 pmol	10 µM	1 h
	11		15 pmol	10 µM	1 h
Bait-SELEX	1	CS3 or CS4	200 pmol	20 µM	2 h
	2		15 pmol	20 µM	2 h
	3		5 pmol	20 µM	2 h
	4		5 pmol	20 µM	1 h
	5		15 pmol	20 µM	1 h
	6		15 pmol	20 µM	1 h

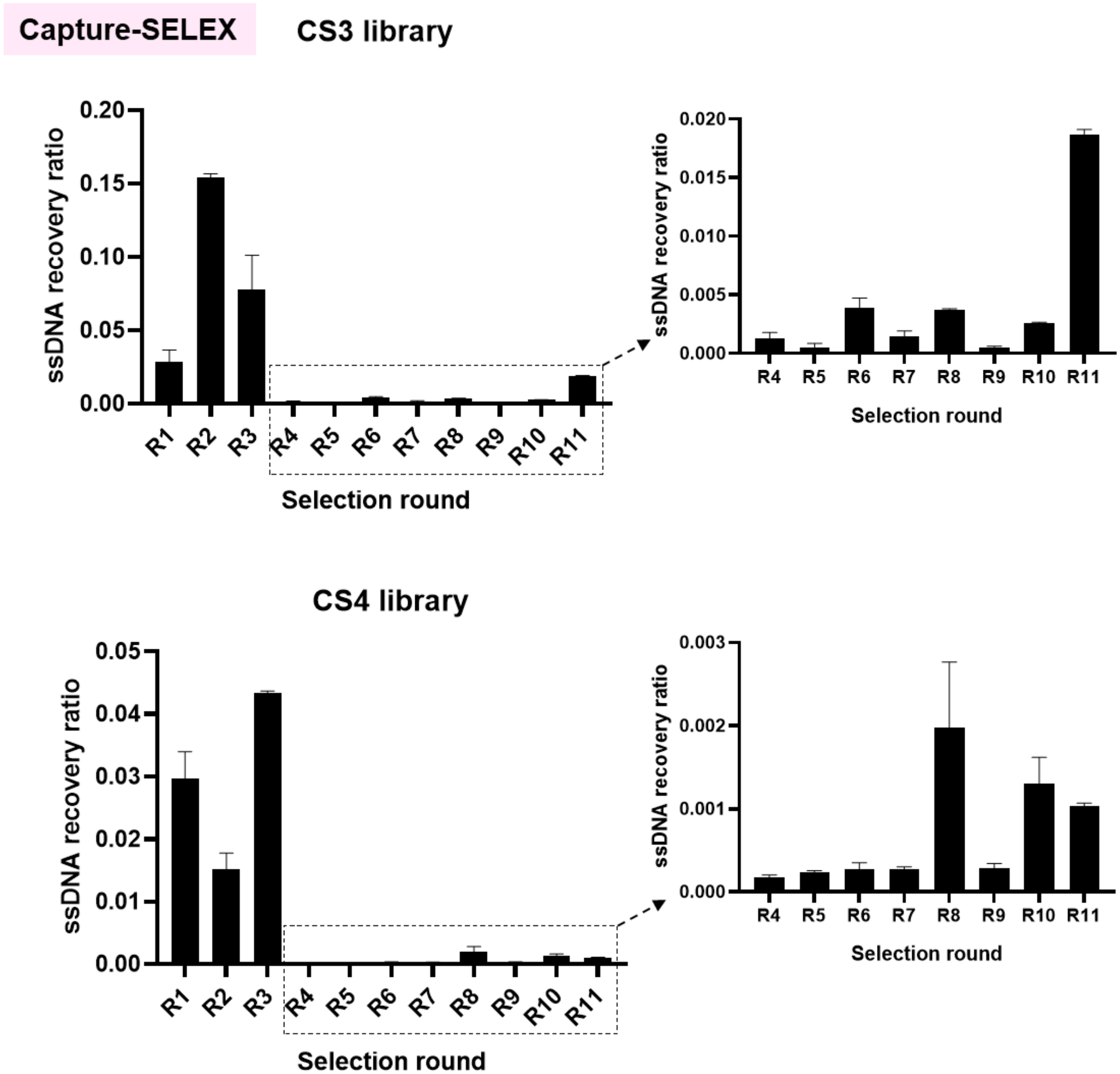

Figure S3. Progress of the capture-SELEX selections monitored by qPCR. The amount of target-bound ssDNA after each round quantified by qPCR was normalized to the input ssDNA library pool in each round.

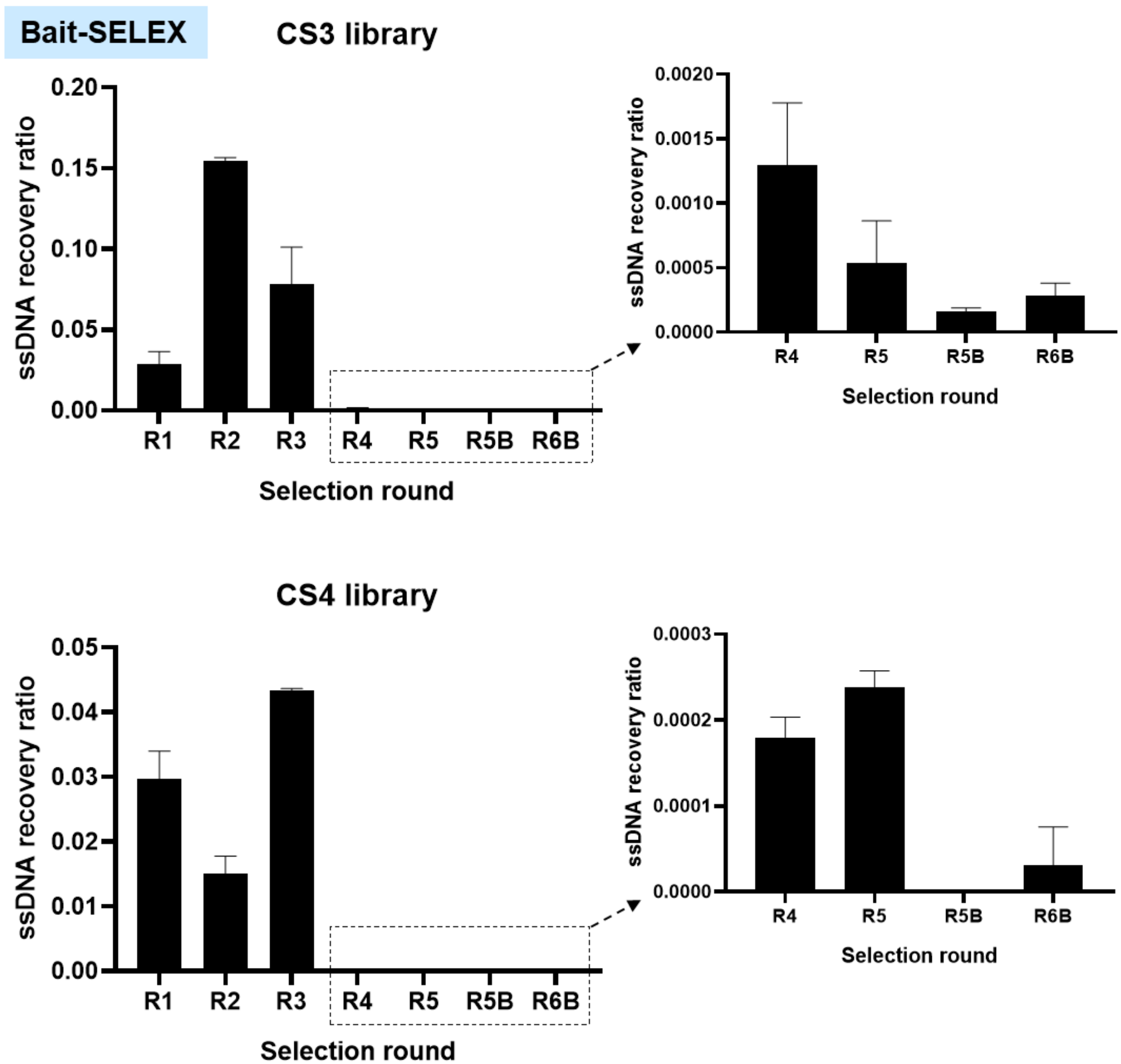

Figure S4. Progress of the bait-SELEX selections monitored by qPCR. The amount of target-bound ssDNA after each round quantified by qPCR was normalized to the input ssDNA library pool in each round.

Table S3. Selected aptamer candidates. The ΔG values for the predicted secondary structures were calculated using the Mfold[1] at SELEX conditions (137 mM NaCl, 1.5 mM $MgCl_2$, 25ºC).

Selection, library	Aptamer	Sequence (5' – 3')	Length (nt)	%GC	ΔG (kcal/mol)
Capture-SELEX, CS3	HC309p	AAGCATCCGCTGGTTGACGAAATATTTTTGAGGCTCGATCCGCCAGGGAGGGAGATCGGGAATGAGATAGGATTAGAGGCATGCCATTTGGGCTGCTC	98	52	-13.89
	HC324p	AAGCATCCGCTGGTTGACGTACTTGAAATGAGGCTCGATCGACCGGAAGCGAGATCGTAAAAAAGAGGCGGCCTGAAAATGATGCCATTTGGGCTGCTC	99	52	-14.79
	HC3104p	AAGCATCCGCTGGTTGACCCCGGGCTCGTGAGACTCGATCAGATAGTCTAAAAACTTTGGACACTAATCAAGAAAAACTTAATGCCATTTGGGCTGCTC	99	46	-12.11
Capture-SELEX, CS4	HC401p	AGCAGTCGTGGCGTAATAATCGGATGGACCCGCTAAATTATAGCTTATCGATCTTGGACCCTGCGAA	67	48	-4.49
	HC403p	AGCAGTCGTGGCGTAATAGTTGATGGATGGACCCGTAAAAACCGACACTGATCTTGGACCCTGCGAA	67	51	-2.06
	HC433p	AGCAGTCGTGGCGTAATAGGCGTATCATGGAAGATAAAGCCCGTAAACGATCTTGGACCCTGCGAA	66	50	-6.71
	HC4106p	AGCAGTCGTGGCGTAATATCACTACCCTGCCCATATTAGAAAGCAGATGATCTTGGACCCTGCGAA	66	48	-5.11
	HC4118p	AGCAGTCGTGGCGTAATAGACCCGCAGTTAAGGAAGCATTAAAGCAGTGATCTTGGACCCTGCGAA	66	50	-5.23
	HC4130p	AGCAGTCGTGGCGTAATAGTCACTACCAAGGAAGGTTAAGTGACCCGCGATCTTGGACCCTGCGAA	66	53	-8.79
	HC4133p	AGCAGTCGTGGCGTAATATATGTTGGATGGACTCAACGTAAGCCCTAAGATCTTGGACCCTGCGAA	66	48	-3.51
Bait-SELEX, CS3	HP301p	AAGCATCCGCTGGTTGACTGAGATGTTATGAGGCTCGATCGAGAAAAGCATATATATTCCGATTAACTGTAGAGTGTTAGATGCCATTTGGGCTGCTC	98	44	-8.70
	HP368p	AAGCATCCGCTGGTTGACTGTAGCATATTGAGGCTCGATCTATAAGATCCGAATAAGTAGGTGGTAATTCAAATTTCAATGCCATTTGGGCTGCTC	96	43	-10.33
	HP389p	AAGCATCCGCTGGTTGACTCAGTATAAGTGAGGCTCGATCGTATGACACGATGAGAAAATTGAGTTATTAGAATTGACTGATGCCATTTGGGCTGCTC	98	44	-13.28
Bait-SELEX, CS4	HP412p	AGCAGTCGTGGCGTAATATTGCTCGTTTACTATAGTATGGAAACTGACGATCTTGGACCCTGCGAA	66	45	-3.19
	HP414p	AGCAGTCGTGGCGTAATAGAAGGCAAGCTTGGTAGCACCTTATTTTAAGATCTTGGACCCTGCGAA	66	47	-4.77
	HP419p	AGCAGTCGTGGCGTAATAGAGTACATAGCCTCCTTAGGATTTGCAAACGATCTTGGACCCTGCGAA	66	48	-3.87
	HP429p	AGCAGTCGTGGCGTAATACAAAATTAACTAAATATTTTAATAGTATGAGATCTTGGACCCTGCGAA	66	35	-1.40

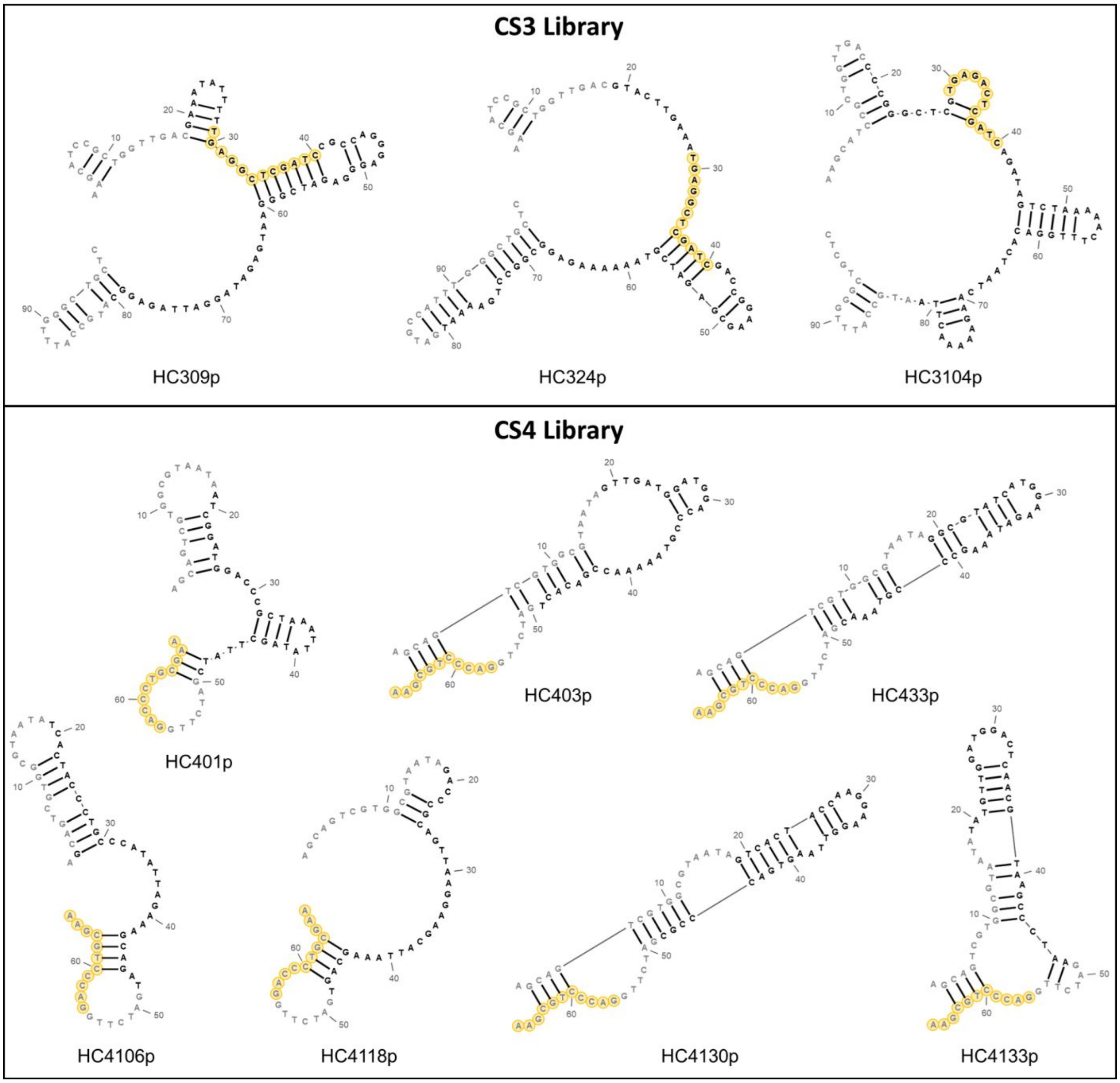

Figure S5. Predicted secondary structures of aptamer candidates from capture-SELEX using Mfold[1] and visualized using RNAcanvas.[2] The docking sites are highlighted in yellow and primer-binding regions are in gray.

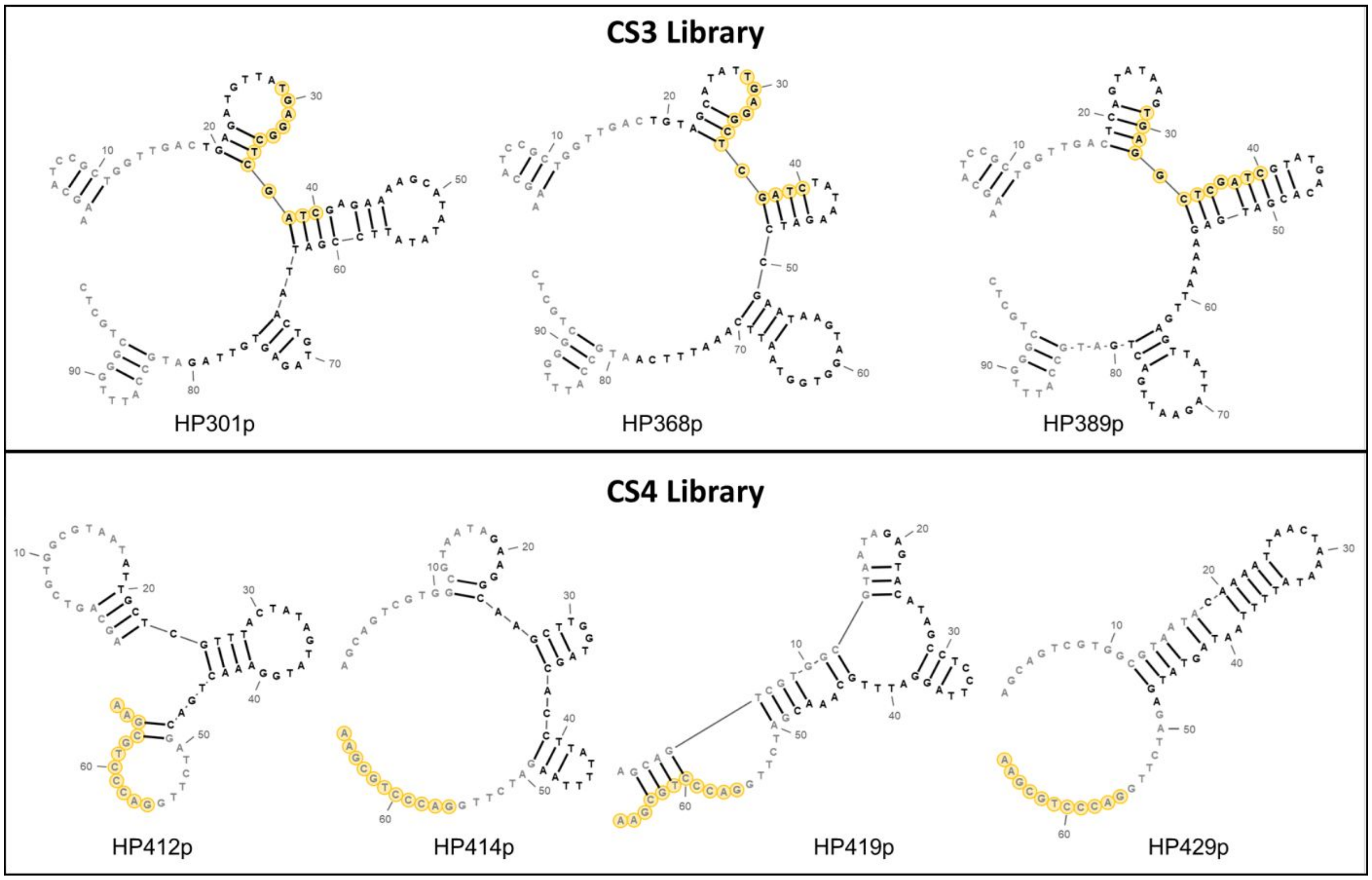

Figure S6. Predicted secondary structures of aptamer candidates from bait-SELEX using Mfold[1] and visualized using RNAcanvas.[2] The docking sites are highlighted in yellow and primer-binding regions are in grey.

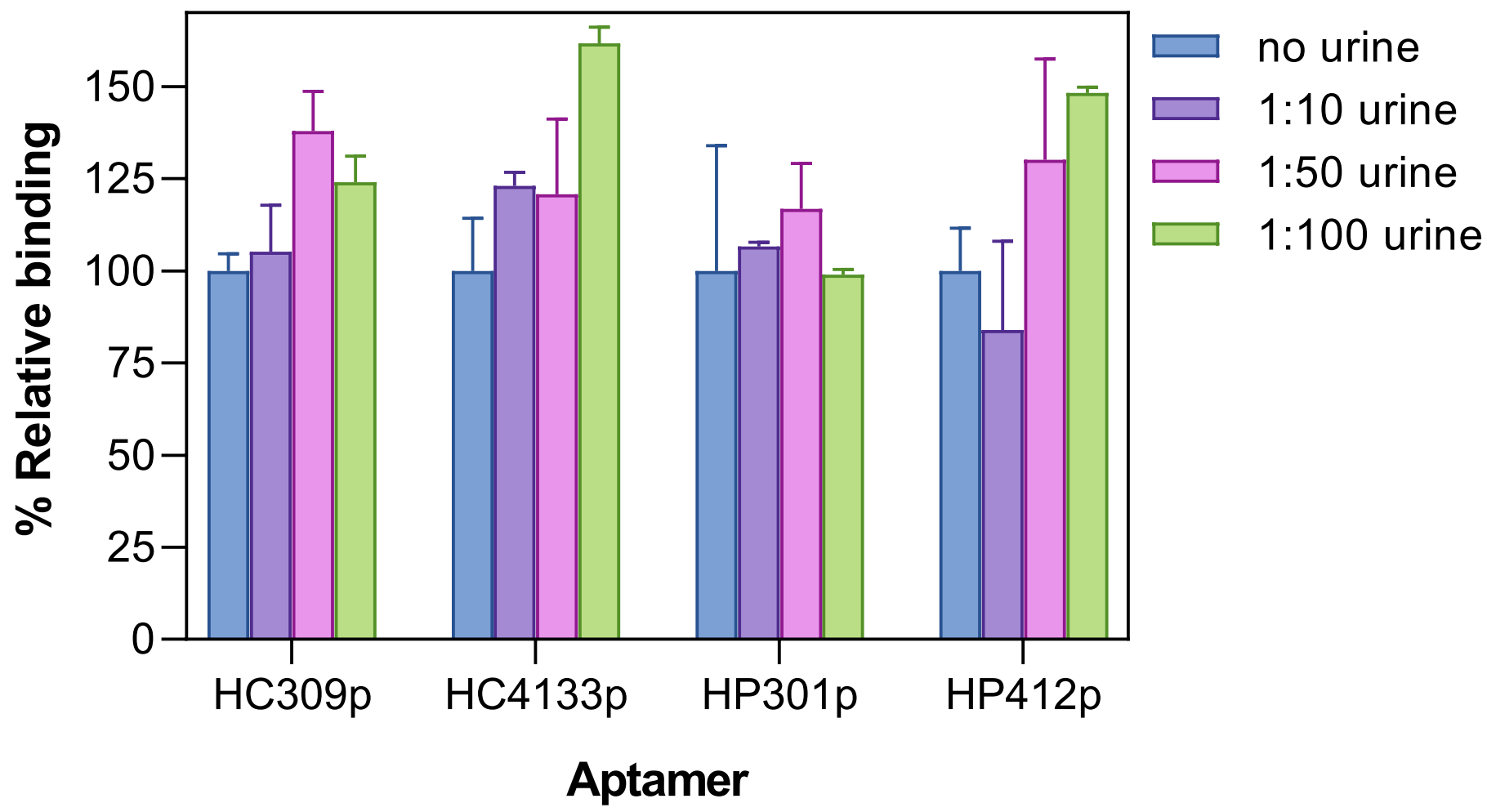

Figure S7. Interactions of the aptamers with ESAT-6/CFP-10 heterodimer in binding buffer spiked with artificial human urine at different dilutions. The signals for the binding of each aptamer to ESAT-6/CFP-10 immobilized on microplate wells in binding buffer were normalized to the signals obtained from their binding in the presence of urine.

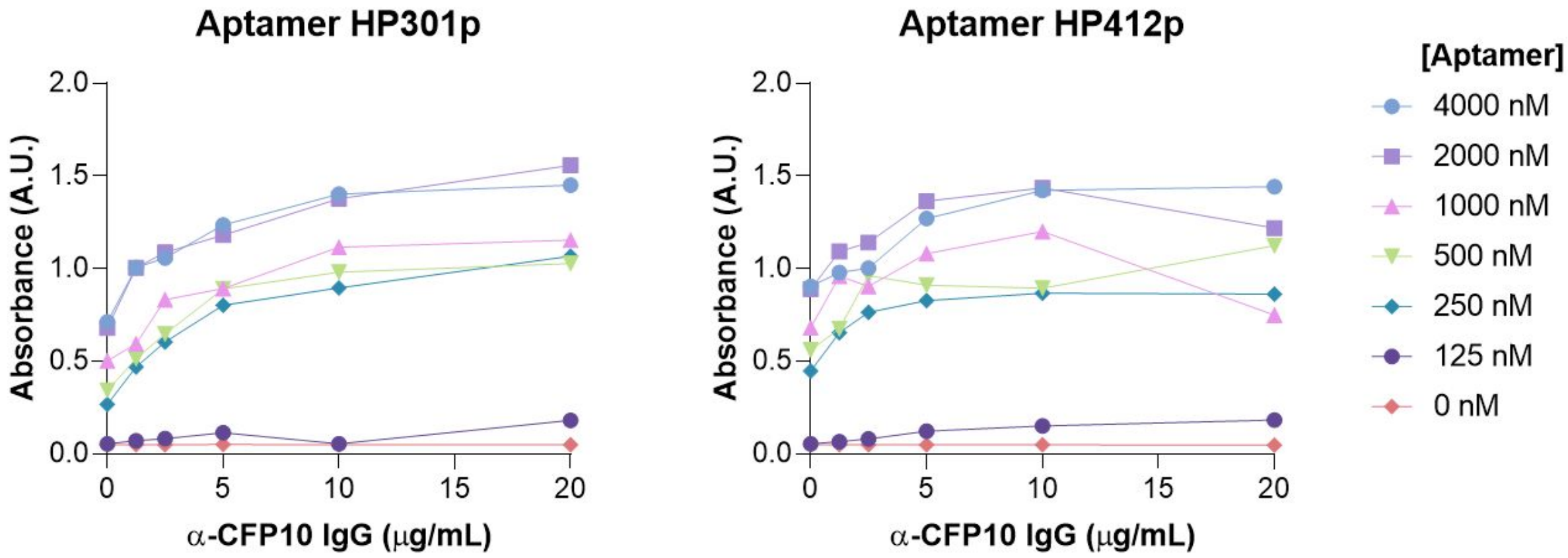

Figure S8. Optimization of the concentrations of the α-CFP-10 IgG antibody (capture) and biotinylated aptamer (detection) in hybrid antibody-aptamer sandwich assay for *M. tuberculosis* ESAT-6/CFP-10 heterodimer detection. The heterodimer was used at 20 μM.

Table S4. Aptamers selected against the *M. tuberculosis* ESAT-6/CFP-10 heterodimeric antigen to date. BLI: biolayer interferometry; CE: single-chain ESAT-6.CFP-10 heterodimer; ELAA: enzyme linked aptamer assay; K_D: affinity dissociation constant; SPR: surface plasmon resonance; TB: tuberculosis.

Selection strategy	Number of rounds	Target	Aptamer	K_D (method); target	Comments	Ref.
Filter membrane SELEX	6	ESAT-6/CFP-10 heterodimer formed *in vitro* by mixing the two monomers at 1:1 molar ratio	CSIR 2.2	21.5 ± 4.3 nM (SPR); CFP-10	Aptamers CSIR 2.11 and 2.21 were applied to indirect ELAA for TB antigen detection in sputum samples	[3]
			CSIR 2.11	8 ± 1.07 nM (SPR); CFP-10		
			CSIR 2.15	12 ± 1.6 nM (SPR); CFP-10		
			CSIR 2.19	1.6 ± 0.5 nM (SPR); CFP-10		
			CSIR 2.21	10.2 ± 3.4 nM (SPR); CFP-10		
Microplate-SELEX	17	Single-chain CE protein with a 15-amino acid linker between the two monomers ESAT-6 and CFP-10	CE15	160 nM (ELAA); CE	Applied in hybrid antibody-aptamer microplate assays for TB antigen detection in serum samples	[4]
			CE24	375 nM (ELAA); CE		
Capture-SELEX	11	ESAT-6/CFP-10 heterodimer formed *in vitro* by mixing the two monomers at 1:1 molar ratio	HC309p	100.2 nM (ELAA), 424 nM (BLI); ESAT-6/CFP-10	-	This work
			HC4133p	273.4 nM (ELAA), 356 nM (BLI); ESAT-6/CFP-10	-	
Bait-SELEX	6		HP301p	232.3 nM (ELAA), 396 nM (BLI); ESAT-6/CFP-10	Applied to antibody-aptamer sandwich microplate assay for heterodimer detection in buffer	
			HP412p	91.8 nM (ELAA), 348 nM (BLI); ESAT-6/CFP-10		

REFERENCES

(1) Zuker, M. Mfold Web Server for Nucleic Acid Folding and Hybridization Prediction. *Nucleic Acids Res.* **2003**, *31* (13), 3406–3415. https://doi.org/10.1093/nar/gkg595.

(2) Johnson, P. Z.; Simon, A. E. RNAcanvas: Interactive Drawing and Exploration of Nucleic Acid Structures. *Nucleic Acids Res.* **2023**, *51* (W1), W501–W508. https://doi.org/10.1093/nar/gkad302.

(3) Rotherham, L. S.; Maserumule, C.; Dheda, K.; Theron, J.; Khati, M. Selection and Application of ssDNA Aptamers to Detect Active TB from Sputum Samples. *PLoS ONE* **2012**, *7* (10), e46862. https://doi.org/10.1371/journal.pone.0046862.

(4) Tang, X.-L.; Zhou, Y.-X.; Wu, S.-M.; Pan, Q.; Xia, B.; Zhang, X.-L. CFP10 and ESAT6 Aptamers as Effective Mycobacterial Antigen Diagnostic Reagents. *J. Infect.* **2014**, *69* (6), 569–580. https://doi.org/10.1016/j.jinf.2014.05.015.
